# Novel magnetically retrievable In_2_O_3_/MoS_2_/Fe_3_O_4_ nanocomposite materials for enhanced photocatalytic performance

**DOI:** 10.1038/s41598-021-85532-8

**Published:** 2021-03-18

**Authors:** Sauvik Raha, Md. Ahmaruzzaman

**Affiliations:** Department of Chemistry, National Institute of Technology Silchar, Silchar, Assam 788010 India

**Keywords:** Pollution remediation, Nanoscience and technology, Nanoscale materials, Magnetic properties and materials

## Abstract

The current work involves synthesis of hybrid nanomaterial of In_2_O_3_/MoS_2_/Fe_3_O_4_ and their applications as photocatalysts for disintegration of esomeprazole under visible light illumination. The data emerged from various analyses testified to the successful construction of the desired nano-scaled hybrid photocatalyst. Tauc plot gave the band gap of In_2_O_3_/MoS_2_/Fe_3_O_4_ to be ~ 2.15 eV. Synergistic effects of the integrant components enabled efficacious photocatalytic performances of the nanocomposite. The nanohybrid photocatalyst In_2_O_3_/MoS_2_/Fe_3_O_4_ showed photodecomposition up to ~ 92.92% within 50 min. The current work realizes its objective of constructing metal oxide based hybrid nano-photocatalyst supported on MoS_2_ sheets for activity in the visible spectrum, which displayed remarkable capacity of disintegrating emerging persistent organic contaminants and are magnetically recoverable.

## Introduction

Water decontamination is currently among the most exhilarating and pioneering fields of science research. For there is a gradual uptick of contamination of water by emerging menaces ranging from dyes to personal care products to pharmaceutical compounds. These emerging, non-biodegradable organic pollutants reach water bodies due to their uncontrolled release from point sources such as factories, plants industries and hospital sewage as well from non-point sources such as runoff. The release of toxic waste filled water into the biosphere brings on its deterioration and undesirable alterations in ecological balance. Among the prominent emerging water pollutants, pharmaceuticals have attracted strong attention of researchers in the field of water purification.

Pharmaceuticals reach water sources through sewage systems carrying the excreta of individuals who have consumed them and from their unregulated disposal. Veterinary pharmaceuticals may be emitted to water either directly as a result of application in fish farming or indirectly via runoff from animal-feeding operations in livestock industry. Pharmaceuticals ridden water exerts tremendous negative impacts on terrestrial and water-dwelling lives. The occurrence of pharmaceutical compounds and their residues and metabolites in wastewater has been an area of growing concern and keen research interest. Esomeprazole, the S-isomer of omeprazole, is a pharmaceutical drug used for the treatment of certain stomach and esophagus problems, such as acid reflux and ulcers. Prominent among its side effects are diarrhea, nausea, constipation, kidney ailment, and autoimmune diseases like, cutaneous lupus erythematosus and systemic lupus erythematosus. Prolonged, interminable use likely lead to a few severe health risks such as vitamin B-12 deficiency, fundic gland polyps, osteoporosis, etc^1–3^. Therefore, the annihilation of esomeprazole in wastewater is of paramount significance.

Among a host of advanced processes for detoxification of wastewater, advanced oxidation processes (AOPs) have seen a surge in application among researchers engaged in investigations geared towards water decontamination. AOPs usually refer to techniques of generating reactive oxygen species (viz., ·OH, ·O_2_^−^, etc.) for thorough destruction of toxic organic substances. Heterogeneous photocatalysis is a type of AOP. It takes on the surface of a semiconductor catalyst. In heterogeneous photocatalysis, the semiconductor particles are excited by photons of energy equal to or greater than the band gap leading to the formation of electron–hole pairs^4,5^. Thereafter, superoxide anion radicals (·O_2_^−^) are formed by the reaction of electrons with oxygen and hydroxyl radicals (·OH) are formed when holes react with water. The energetic radicals thus generated then make indiscriminate onslaughts on the molecules of the organic contaminant breaking them down to less persistent, innocuous organic compounds and simple end products such as CO_2_, H_2_O, etc. Superior detoxification ability, inexpensive operation and modest operational conditions of experiment together impart semiconductor photocatalysis a high ground in tackling water contamination. Enormous surface area, chemical stability and size-dependent properties make nano-scaled semiconductor materials apt for application in water decontamination^6^. Among several semiconductor nanomaterials, TiO_2_ has attracted attention as efficient, photo-chemically stable and ecofriendly photocatalyst vis-à-vis decomposition of organic pollutants^7,8^. ZnO, WO_3_, CeO_2_, In_2_O_3_, Nb_2_O_5_, Ta_2_O_5_, MOS_x_, WS_x_, CdS, ZnS, etc. have also been recently used for photocatalysis^9–11^. On one hand, narrow semiconductor materials are beset with severe recombination of photo-induced charge carriers, while on the other hand, semiconductors possessing large band gap primarily absorb in the ultra violet region and are therefore incapable of utilizing a large portion of the solar light rendering them exclusively UV active. This has driven development of strategies that rely upon integration of wide and narrow band gap semiconductors for fine tuning band edges and enabling absorption of photons in the visible spectrum while also preventing the rapid recombination of electron and holes. Such integrated photocatalysts have displayed superlative performance in the photodecomposition of organic water contaminants. MoS_2_/TiO_2_^12^, Ag_3_PO_4_/TiO_2_/MoS_2_^13^, Bi_2_O_3_/Bi_2_S_3_/MoS_2_^14^, etc. have recently been designed for application in photocatalytic decomposition of emerging organic water pollutants. Additionally to facilitate easy and inexpensive recovery of photocatalysts from reactors, Fe_3_O_4_ has been used as the superparamagnetic component in coupled photocatalysts^15–17^. Fe_3_O_4_ has also been known to step up photodecomposition via charge separation through trapping of light-generated electrons by Fe^3+^ ions^18,19^. Besides, recently, Fe as a component in electrocatalysts has been reported to play a key role in triggering oxygen evolution reaction (OER)^20^. The authors of the current work have thus designed an integrated system of photocatalyst that responds in the visible spectrum, is photo-chemically stable, bears the capacity to disintegrate recalcitrant organic pollutants and is magnetically recoverable. Indium oxide (In_2_O_3_) is an important n-type semiconductor. It has three phases, namely, hexagonal, cubic and hexagonal corundum. Of late, In_2_O_3_ has seen wide applications in solar cells, sensor modules, transparent electrode materials for both electrochromic cells and for liquid crystal display devices, flat-panel displays, light-emitting diodes, thin-film transistors, etc^21,22^. Chief advantages of In_2_O_3_ for use in photocatalysis include high photosensitivity, environmental stability and it being an efficient sensitizer to extend the absorption spectra of metal oxide-based semiconductor photocatalysts from the UV spectrum into the visible spectrum. Its drawback is a rapid recombination of photo-induced electrons and holes^21,22^.

Coupling In_2_O_3_ with Fe_3_O_4_ would allow fabrication of a magnetically retrievable photocatalyst besides facilitating charge separation through electron trapping by Fe^3+^ ions. Loading In_2_O_3_/Fe_3_O_4_ onto a semiconducting material that could act as support and an integrating moiety for band gap tailoring that ensure further elongation of the lifetime of charge carriers would result into the construction of a visible light active superparamagnetic photocatalyst with superior stability. Therefore, In_2_O_3_/Fe_3_O_4_ was loaded MoS_2_ sheets for the fabrication In_2_O_3_/MoS_2_/Fe_3_O_4_ hybrid nano-scaled photocatalyst. Molybdenum disulfide (MoS_2_), a kind of two-dimensional layered transition metal dichalcogenide, has unique advantages in photocatalysis in view of its unique physical, chemical and electrical properties and tunable band structure^23,24^. MoS_2_ matrix prevents agglomeration of nanoparticles. Also MoS_2_ has of late been employed as a cocatalyst in photocatalysis induced decontamination of water^25^. Facile procedure of synthesis was adopted in the current work for construction of In_2_O_3_/MoS_2_/Fe_3_O_4_ hybrid nano-scaled photocatalyst. The ternary nano-scaled photocatalyst was found to exhibit excellent photocatalytic activities disintegrating esomeprazole, the target pollutant, within 50 min. The photodecomposition of esomeprazole by this nano-photocatalyst was found to follow pseudo-first order kinetics. In_2_O_3_/MoS_2_/Fe_3_O_4_ attained 92.92 ± 2.01% photodecomposition at a velocity constant of 0.06208 min^−1^ with TOC and COD reduction up to ~ 77.06% and ~ 66.71%. The fabrication of novel ternary nano-photocatalyst with excellent capacity to disintegrate an emerging water contaminant, esomeprazole, with superlative velocity constants marks the novelty of this work.

## Experimental details

### Materials and measurement

The following items of AR grade were acquired from Sigma Aldrich and used without further purification: Indium (III) nitrate hydrate (In(NO_3_)_3_.xH_2_O), sodium hydroxide (NaOH), ferrous sulphate heptahydrate (FeSO_4_ 0.7H_2_O), ferric chloride (FeCl_3_), molybdenum(IV) sulfide (MoS_2_), esomeprazole magnesium, deionized water etc.

Most decomposition studies are performed in pure water ignoring the effects of various forms of environmental water on photodecomposition. For better understanding of photocatalysis, it is worth investigating the influences of the attendance of various inorganic ions, organic substances and various water matrices on the performances of the fabricated photocatalysts. A few grab samples of environmental waters namely mineral water, tap water and river water were therefore acquired for the investigation. Packaged sachet mineral water with pH 7.3 and TOC 0.25 ppm was procured from a local supermarket. Tap water with pH 7.8 and TOC 3.5 ppm was obtained from local drinking water systems while river water sample was sourced from the Barak River flowing through the state of Assam in India. The grab river water sample had a pH of 8.34 and TOC 5.3 ppm.

### Synthesis

#### Synthesis of In_2_O_3_/MoS_2_/Fe_3_O_4_ and pristine samples

Deionized water was taken in a beaker and to it were added 20 mmol of FeSO_4_ 0.7H_2_O and 30.8 mmol of FeCl_3_. Magnetic stirrer was used for dissolving the salts. Thereafter, aqueous solution of sodium hydroxide (NaOH) (224.8 mmol) was slowly added to it. The reaction mixture was left for stirring for 18 h. Next, 20 mmol of Indium (III) nitrate hydrate (In(NO_3_)_3_.xH_2_O) was added to this reaction mixture followed by stirring. An aqueous solution of sodium hydroxide (NaOH) (30 mmol) was then slowly poured into it. The final reaction mixture was stirred and then taken in a Teflon autoclave maintained at 110 °C for 18 h. The precipitate thus obtained was centrifuged and washed thoroughly with ethanol. After collection, the sample was dried and thereafter annealed at 300 °C for 3 h. The brown sample thus obtained was treated as In_2_O_3_/Fe_3_O_4_.

Next, MoS_2_ was exfoliated by ultrasonication in distilled water following which In_2_O_3_/Fe_3_O_4_ was added to it. This mixture was also ultrasonicated for 5 min and then dried to obtain the final nanohybrid of In_2_O_3_/MoS_2_/Fe_3_O_4_.

Pristine samples of In_2_O_3_, Fe_3_O_4_, exfoliated MoS_2_ and the binary nanohybrid were likewise prepared following the procedure illustrated above but only in presence of the respective starting materials. Indium (III) nitrate hydrate (In(NO_3_)_3_.xH_2_O) was used for fabricating In_2_O_3_ while Fe_3_O_4_ was prepared using the aforementioned iron salts in aforesaid proportion.

#### Characterization

In_2_O_3_/MoS_2_/Fe_3_O_4_, In_2_O_3_/Fe_3_O_4_, In_2_O_3_, MoS_2_, and Fe_3_O_4_ were analyzed for X-ray diffraction studies on Phillips XPERT powder X-ray diffractometer with Cu-K_α_ radiation. TEM, HRTEM and SAED were executed on JEOL JEM 2100 instrument for determination of size, morphology and diffraction rings of In_2_O_3_/MoS_2_/Fe_3_O_4_. X-ray photoelectron spectroscopy (XPS) of the final nanocomposite was carried out with PHI 5000 Versa Prob II spectrometer to investigate the electronic environment and valence states of elements in the two ternary nanocomposites. Photoluminiscence data were acquired with Hitachi F4600 equipment. Magnetic behaviour of Fe_3_O_4_ and In_2_O_3_/MoS_2_/Fe_3_O_4_ was ascertained employing Vibrating Sample Magnetometer. HRLCMS graphs were obtained using 1290 Infinity UHPLC System, Agilent Technologies, USA. TOC results were collected following analyses carried out using Elementar, Liqui TOC. GENESYS 10S UV–visible spectrophotometer was used for obtaining the absorbance spectra of various samples.

#### Evaluation of photocatalytic performance

The esomeprazole photodecomposition experiments were carried out in reaction cells that had working volume of 100 mL. LED light irradiation (with 11,770 lx and radiation intensity of 47.14 Wm^−2^) from an LED bulb affixed atop (Philips 23 W white LED) was shone over the reaction cell within a homemade chamber. A luxmeter was employed for the measurement of lux and radiation intensity. In a typical photocatalytic experiment, specified amount of the photocatalyst (0.7 gL^−1^ of In_2_O_3_/MoS_2_/Fe_3_O_4_) is suspended in 50 mL of aqueous solution of esomeprazole prepared using distilled water with at an initial pH of 5. Other requisite substances were incorporated before the dispersal of the photocatalyst in order to simulate aqueous matrices. Water matrices replaced distilled water in other investigative studies about the effect of environmental water samples on photodecomposition. Each test was carried out in quintet, putting the suspension in a dark environment under agitation for 30 min to attain adsorption–desorption equilibrium followed by exposure to LED illumination for 50 min for photodecomposition. The photodecomposition was recorded by noting the absorbance at the λ_max_ of esomeprazole at 301 nm after every 10 min.

The photodecomposition efficiency was calculated using the following equation:

1 $$Degradation\;efficiency \left( \% \right) = \left( {\frac{{C_{0} - C}}{{C_{0} }}} \right) \times 100$$

where the initial concentration of esomeprazole and the concentration after time t are given by C_0_ and C.

The photodecomposition kinetics was assessed using the following equation:

2 $$ln\frac{{C_{0} }}{C} = kt$$

where the initial concentration of esomeprazole and the concentration after time t are given by C_0_ and C and k denotes the velocity constant of the photodegradation reaction proceeding in accordance with pseudo-first order kinetics model.

The error bars in the photodecomposition diagrams represent small standard deviations suggesting fair reproducibility of the experiment for five repetitions under a given set of conditions.

## Results and discussion

### XRD studies

**Figure 1:**
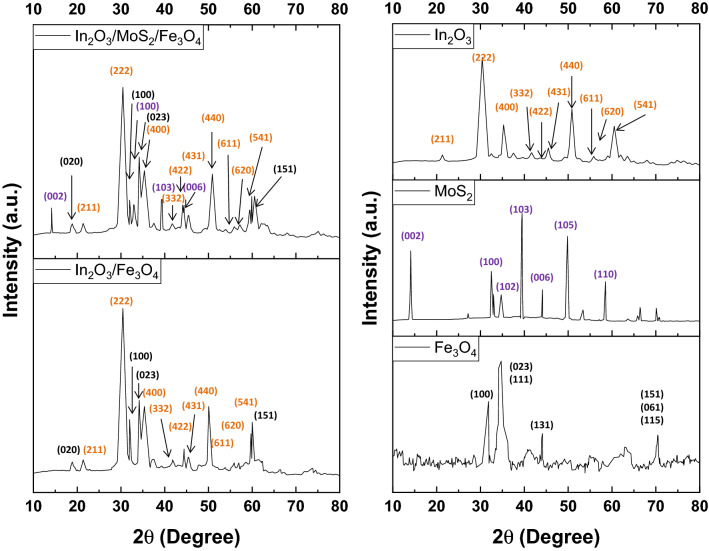
XRD patterns of different samples of In_2_O_3_/MoS_2_/Fe_3_O_4_, In_2_O_3_/Fe_3_O_4_, In_2_O_3_, MoS_2_ and Fe_3_O_4_.

The XRD diffraction pattern of In_2_O_3_/MoS_2_/Fe_3_O_4_ (Fig. 1) recorded prominent peaks corresponding to (222), (400) and (440) planes cubic In_2_O_3_ phase (JCPDS 06–0416) at 2θ angles of 30.63°, 35.52° and 51.06° respectively^11^ besides displaying peaks that could be indexed to (422), (431), (611), (620) and (541) planes at 2θ angles of 21.66°, 41.97°, 43.84°, 45.91°, 56.07°, 57.68° and 59.27° respectively. The presence of a prominent (023) peak at 34.23° along with accompanying peaks of (020), (100) and (151) planes at 18.96°, 32.08° and 60.09° respectively confirmed Fe_3_O_4_ phase (JCPDS 89–6466) in the nanohybrid. Furthermore, MoS_2_ phase (JCPDS 73–1508) could be ascertained by peaks indexed to (002), (100), (102), (103) and (006) planes at 14.45°, 32.86°, 35.21°, 39.72° and 44.34° respectively. The average crystallite size obtained using Scherrer formula in the ternary nanohybrid, In_2_O_3_/MoS_2_/Fe_3_O_4_, was 13.47 nm. In the powder XRD pattern of In_2_O_3_/Fe_3_O_4_ (Fig. 1), the prominent peaks of In_2_O_3_ (JCPDS 06–0416) and Fe_3_O_4_ (JCPDS 89–6466) phases could be identified. Peaks associated with (222), (400) and (440) planes of the cubic In_2_O_3_ phase at 2θ angles of 30.62°, 35.50° and 51.04° respectively along with those corresponding (211), (332), (422), (431), (611), (620) and (541) planes at 2θ angles of 21.62°, 41.90°, 43.79°, 45.81°, 56.02°, 57.61° and 59.19° respectively showed up^11^. Also were present peaks indexed to (020), (100), (023), and (151) planes at 18.96°, 32.08°, 34.21° and 60.09° respectively of Fe_3_O_4_ phase (JCPDS 89–6466) in the XRD plot of In_2_O_3_/Fe_3_O_4_. The average crystallite size obtained using Scherrer formula in the binary nanohybrid, In_2_O_3_/Fe_3_O_4_, was 13.78 nm. The XRD plots of the ternary and binary nanocomposites have been reproduced with the corresponding JCPDS cards of the pristine samples in Figure S1 (ESI). The XRD plot of the pristine In_2_O_3_ sample (Fig. 1) demonstrated (222), (400) and (440) planes of cubic In_2_O_3_ phase (JCPDS 06–0416) at 2θ angles of 30.56°, 35.42° and 51.01° respectively^19^ as well as other peaks (211), (332), (422), (431), (611), (620) and (541) planes at 2θ angles of 21.57°, 41.86°, 43.78°, 45.79°, 55.97°, 57.54° and 59.14° respectively. The average crystallite size obtained using Scherrer formula of the nano-scaled In_2_O_3_ was 14.41 nm. The XRD plot of pristine MoS_2_ (Fig. 1) showed (002), (100), (103), (006), (105) and (110) planes of MoS_2_ phase (JCPDS 73–1508) at 14.39°, 32.80°, 39.65°, 44.15°, 49.88° and 58.58° respectively^26^. The powder XRD pattern of Fe_3_O_4_ (Fig. 1) displayed a distinct peak at 30.33° corresponding to (100) plane (JCPDS 89–6466). It was additionally characterized by the occurrence of a superimposition of peaks associated with (023) and (111) planes with respective 2θ values of 34.15° and 34.72°. The plot bore four other minor peaks associated with (131) (115), (061) and (151) planes at 44.38°, 59.65°, 59.65° and 59.98°. The average crystallite size obtained using Scherrer formula of the nano-scaled Fe_3_O_4_ was 17.56 nm.

### TEM, HRTEM and SAED analyses

**Figure 2:**
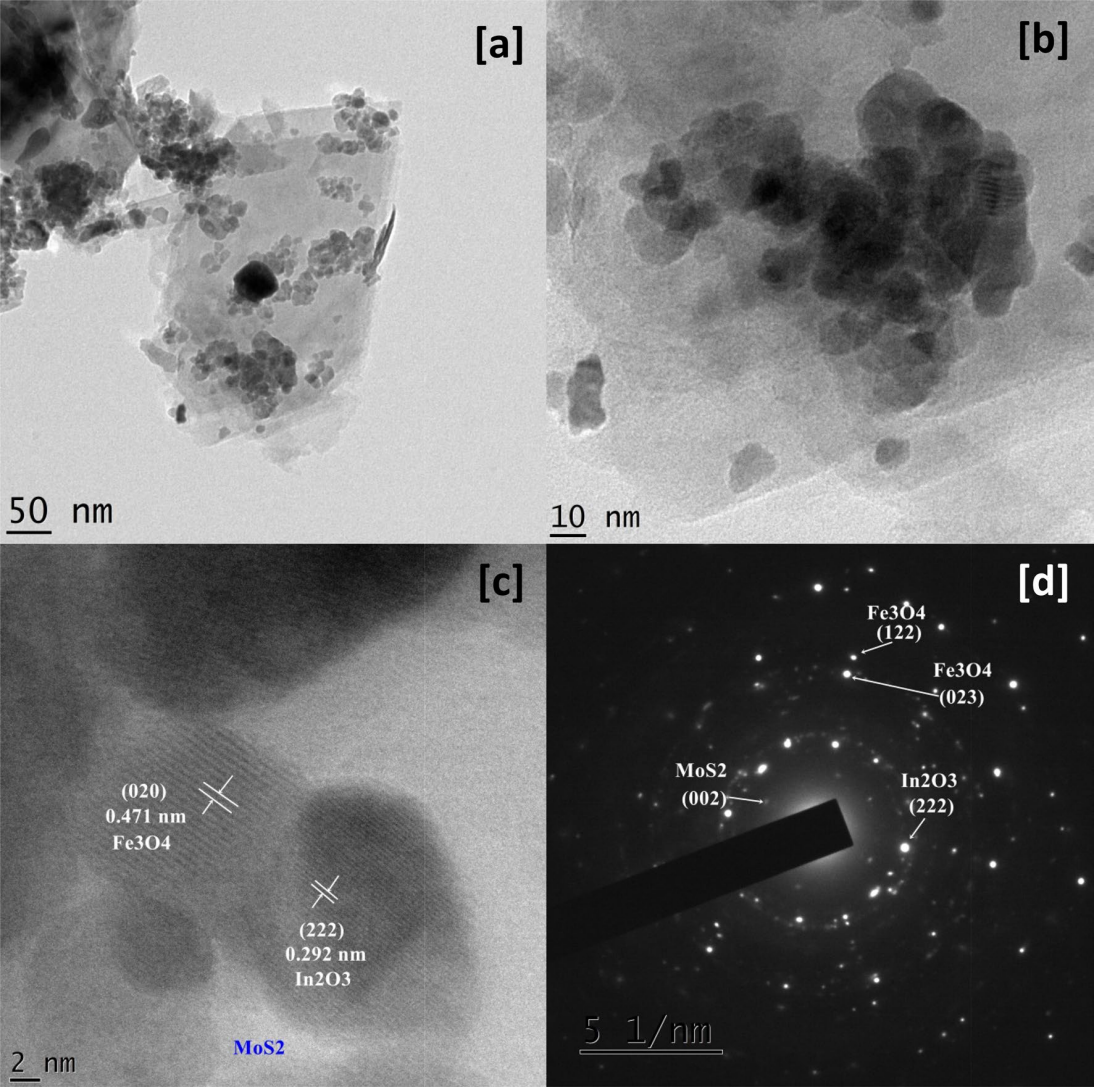
TEM (**a**,**b**) micrographs, HRTEM (**c**) micrograph and SAED patterns (**d**) of In_2_O_3_/MoS_2_/Fe_3_O_4_.

**Figure 3:**
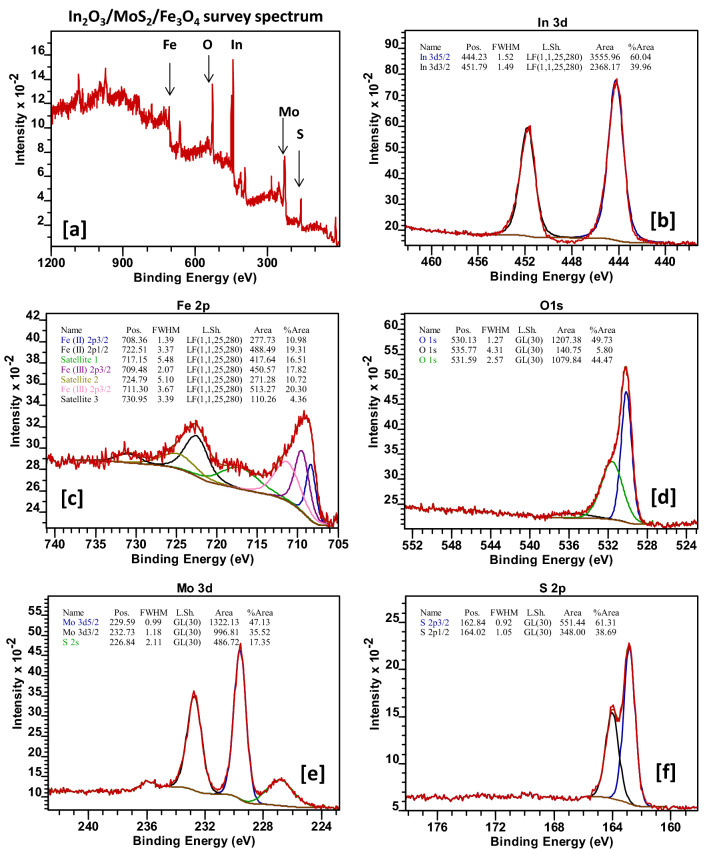
(**a**) XPS survey spectrum of In_2_O_3_/MoS_2_/Fe_3_O_4_, HR-XPS spectra of (**b**) In, (**c**) Fe, (**d**) O, (**e**) Mo and (**f**) S in In_2_O_3_/MoS_2_/Fe_3_O_4_.

The TEM micrographs of In_2_O_3_/MoS_2_/Fe_3_O_4_ (Fig. 2a,b) showed nano-scaled particles of In_2_O_3_ and Fe_3_O_4_ strewn across nanosheet of MoS_2_. The average diameter of these particles was found to be 13.94 nm. From the HRTEM image (Fig. 2c), distinct lattice fringes were revealed. Interplanar spacings allowed recognition of In_2_O_3_ and Fe_3_O_4_ nanoparticles over sheet of MoS_2_. The spacing of 0.292 nm could be linked to the (222) plane of cubic-phased In_2_O_3_ particle^11^ while that of 0.471 nm could be linked to (020) plane of Fe_3_O_4_. Although MoS_2_ nanosheet could not be clearly detected in the higher 2 nm resolution, a slightly lower 5 nm resolution image of the same region revealed flakes of MoS_2_ having a crystalline phase showing facet (002) that could be recognized from an interplanar spacing of 0.61 nm (Figure S2a ESI). The polycrystalline nature of the nanohybrid was suggested by the presence of concentric rings in the SAED pattern (Fig. 2d). Concentric rings corresponding to (222) plane of In_2_O_3_; the (122) and (023) planes of Fe_3_O_4_ and the (002) plane of MoS_2_ nanosheet were identified and marked in the SAED pattern (Fig. 3d).

### XPS investigation

For proper evaluation of the chemical composition and oxidation state of elements in the synthesized nanohybrid of In_2_O_3_/MoS_2_/Fe_3_O_4_, the sample was examined using XPS analysis. The XPS survey spectrum of In_2_O_3_/MoS_2_/Fe_3_O_4_ (Fig. 3a) displayed peaks associated with In 3d, Fe 2p, O 1 s, Mo 3d and S 2p. The 3d core level spectrum of In (Fig. 3b) revealed two peaks at 444.23 eV and 451.79 that corresponded to 3d_5/2_ and 3d_1/2_ electrons of In^3+^ ^27^. The reported values of binding energy of 3d_5/2_ and 3d_1/2_ electrons of In^3+^ hover around 444 eV and 451.50 eV^27^. This suggests a small shift towards higher binding energy values of 3d_5/2_ and 3d_1/2_ orbitals of In^3+^ in the ternary nanocomposite. The HR-XPS of Fe (Fig. 3c) produced a doublet centered on binding energy values of 710 eV and 722 eV corresponding to Fe 2p_3/2_ and Fe 2p_1/2_. Deconvolution further unveiled contributions from Fe^2+^ in octahedral environment and Fe^3+^ ions in either octahedral or tetrahedral sites^28–31^. Peaks at 708.36 eV and 722.51 eV could be assigned to Fe^2+^ 2p_3/2_ and Fe^2+^ 2p_1/2_ electrons while peaks at 709.48 eV and 711.30 eV could be associated with the Fe^3+^ ion encased within octahedral and tetrahedral voids respectively. The core level spectrum was also characterized by the presence of three satellite peaks at binding energies of 717.15 eV, 724.79 eV and 730.95 eV^28–31^. These binding energy values had slightly lower magnitude in pristine samples of Fe_3_O_4_ as reported in previous works^28,29^. The high-resolution O 1 s spectrum (Fig. 3d) contained three peaks positioned at 530.13 eV, 531.59 eV and 535.77 eV that, in all likelihood, were generated lattice bound O^2−^ ions, O^2−^ ion in the oxygen-vacancy zone and chemisorbed oxygen on the nanohybrid surface respectively. Figure 3e contains core level signals of Mo 3d and S 2s^26,32,33^. The Mo 3d was found to be doublet consisting of 3d_5/2_ at 229.59 eV and 3d_3/2_ at 232.73 eV^26,32,33^. The S 2s signal appears at 226.84 eV^32,33^. The S 2p core level spectrum (Fig. 3f) was marked by the presence of a doublet at binding energy values of 162.84 eV and 164.04 eV corresponding to S 2p_3/2_ and S 2p_1/2_ electrons^32,33^. However, in pristine sample reported in literature^26,32,33^, all these peaks took on slightly lower binding energy values. The abovementioned facts, therefore suggest a successful synthesis of a coupled nano-scaled semiconductor system, namely, In_2_O_3_/MoS_2_/Fe_3_O_4_. The slight shift in binding energy values observed in core level spectrum of individual elements in comparison with those in pristine samples of previously reported works could be ascribed to the creation of a different electron density arising from transfer of electrons and interactions at the interface caused by the formation of a heterojunction in the nanocomposite^34^.

### SEM and EDAX studies

**Figure 4:**
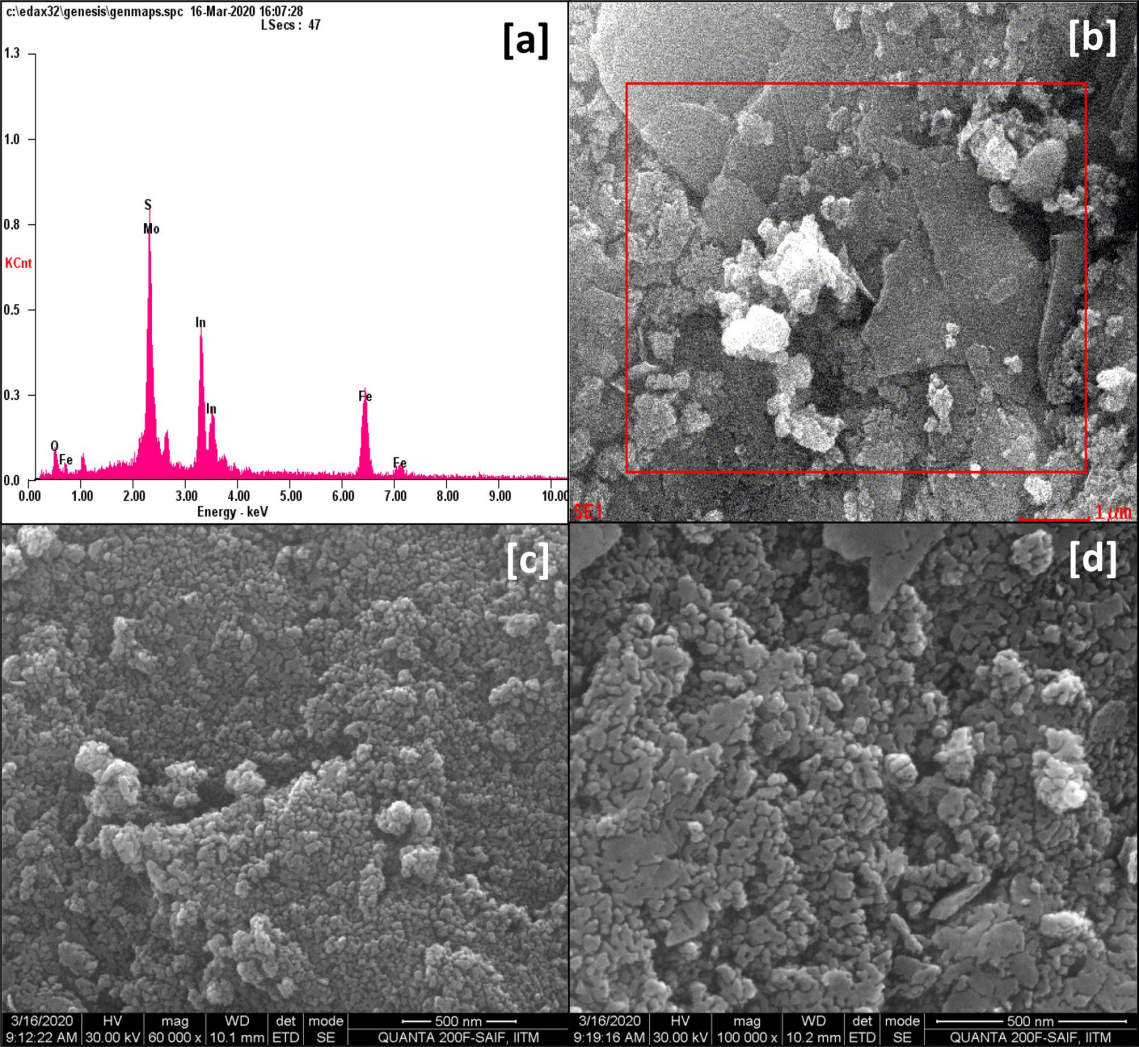
(**a**) EDAX spectrum of In_2_O_3_/MoS_2_/Fe_3_O_4_. (**b**), (**c**) and (**d**) SEM micrographs of In_2_O_3_/MoS_2_/Fe_3_O_4_.

SEM micrographs (Fig. 4b,c,d) of the nanohybrid again showed nanoparticles of the two metal oxides spread across sheets of MoS_2_. MoS_2_ nanosheets were discernible in Figure S2b (ESI). Furthermore, more significant information was extracted from EDAX analysis of In_2_O_3_/MoS_2_/Fe_3_O_4_. From the EDAX spectrum (Fig. 4a), Table 1 presenting the elemental composition of the nanohybrid was made. The EDAX spectrum In_2_O_3_/MoS_2_/Fe_3_O_4_ showed the K_α_ lines of Fe and O, at 6.4 eV and 0.50 eV. L_α_ lines of In and Fe at 3.30 eV and 0.70 eV were also visible. In_2_O_3_/MoS_2_/Fe_3_O_4_ EDAX spectrum additionally bore the L_α_ line of Mo and K_α_ emission of S both standing superimposed at 2.30 eV. The SEM-EDAX analyses therefore lend credence to the successful formation of the desired nanohybrid photocatalyst.

**Table 1:** A chart of the elemental composition of the nanohybrid In_2_O_3_/MoS_2_/Fe_3_O_4_ from EDAX spectrum.

Element	Line Type	Weight%	Atomic%
O	K-series	9.97	32.84
In	L-series	37.44	17.18
Fe	K-series	23.61	22.28
Mo	L-series	18.20	9.99
S	K-series	10.78	17.71
Total		100	100

### Investigation of optical properties

**Figure 5:**
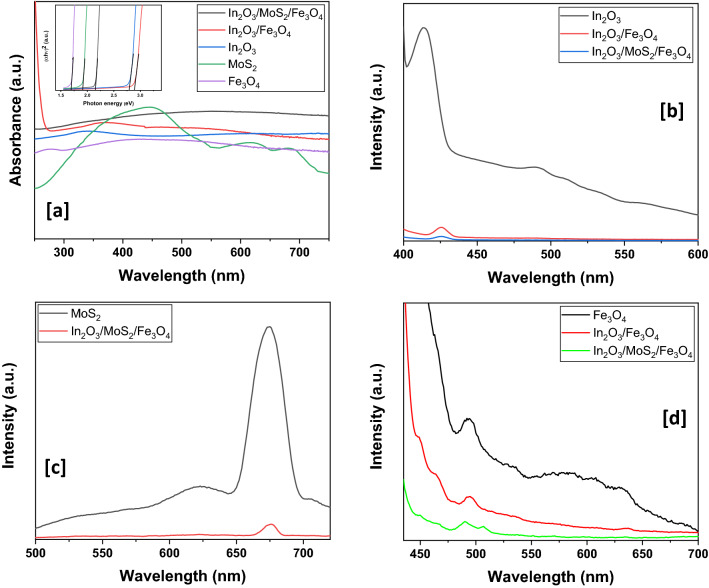
(**a**) UV–visible absorbance spectra of the various nanomaterials with an inset showing their respective Tauc’s plot for calculation of band gaps and (**b**,**c**,**d**) Photoluminiscence graphs.

For evaluation of optical performance of the prepared nano-scaled samples, they were subjected to UV–visible spectrometry (Fig. 5a). The absorption edges of pristine In_2_O_3_, and Fe_3_O_4_ were found to be around ~ 344 nm^35^ and ~ 424 nm respectively generating from transition of electrons from the valence band to the conduction band. The hump of the Fe_3_O_4_ absorbance curve is readily evident in the enlarged picture of Figure S3a (ESI). The UV–vis absorption spectra of pristine MoS_2_ (Fig. 5a) revealed a broad absorption peak centered on ~ 450 nm owing to direct excitonic transitions at the point K in the Brillouin zone^36,37^. On the other hand, peaks at ~ 620 nm and ~ 678 nm could be arising from excitonic transitions from the deep level in the valence band to the conduction band at the M point of the Brillouin zone^36,37^. The absorption maxima of the binary nanohybrid In_2_O_3_/Fe_3_O_4_ showed an extended hump centered on ~ 363 nm. Coupling could have resulted in a red shift from the absorption maxima of the pristine In_2_O_3_. The UV–visible spectra of final nanocomposite of In_2_O_3_/MoS_2_/Fe_3_O_4_ revealed hump centered at ~ 520 nm extending across the visible region. Probable electronic transitions from valence band to conduction band of the ternary nanohybrids and besides substantial interfacial interactions among the moieties of the nanohybrids are thus indicated. Additionally, there could also arise mixing of the outermost s orbitals of In and Fe creating conduction bands at lower energy values. Tauc’s plot (inset of Fig. 7a) was employed to evaluate the band gaps of all the synthesized samples. Pristine In_2_O_3_, Fe_3_O_4_, and MoS_2_ had band gaps of 2.79 eV, 1.70 eV and 1.91 eV, respectively. Enlarged diagram of Tauc’s plot is provided in Figure S3b (ESI). The band gap of In_2_O_3_/Fe_3_O_4_ was found to be 2.88 eV. The final nanohybrid of In_2_O_3_/MoS_2_/Fe_3_O_4_ had band gap of 2.15 eV. UV–visible absorbance data confirmed the integration of different moieties in the formation of the intended nanohybrid.

Photoluminiscence (PL) spectroscopy was carried out to access further insights into the optical properties and to make a comparison of the photoluminiscence behaviour of the as-fabricated nanohybrid samples with the pristine moieties. The emission spectrum of In_2_O_3_ (Fig. 5b) is obtained by exciting the sample at ~ 344 nm. A strong blue emission band around ~ 413 nm marked the spectrum. A small peak was observed at ~ 490 nm due to red emission^38^. The intensity of the emission peak decreased with an increase of doping concentration. A near absence of these emission peaks in the photoluminescence spectra of the nanohybrids suggested a decline in the rate of charge recombination. Further work is to be done in order to grasp a definite insight into the phenomenon. The PL spectrum of pristine MoS_2_ (Fig. 5c) recorded peaks at ~ 624 nm and ~ 674 nm, associated with the exciton transitions in the K-point of the Brillouin zone, respectively^36,37,39–41^. The emission peaks at ~ 623 nm and ~ 674 nm are characteristic exciton peaks of MoS_2_ monolayer whose energy separation is due to spin–orbit splitting at the top of the valence band at the K point of the 1st Brillouin zone^41^. The emission spectrum of In_2_O_3_/MoS_2_/Fe_3_O_4_ recorded a peak at ~ 674 nm with a greatly diminished intensity. Finally, comparison of In_2_O_3_/MoS_2_/Fe_3_O_4_ was made with the pristine Fe_3_O_4_ sample and for this purpose all the samples of In_2_O_3_/MoS_2_/Fe_3_O_4_, In_2_O_3_/Fe_3_O_4_ and pristine Fe_3_O_4_ were excited at ~ 421 nm and the consequent emission spectra were duly registered (Fig. 5d). A sharp peak at ~ 490 nm characterized the Fe_3_O_4_ PL spectrum. Minor peaks cluttered around ~ 575 nm, ~ 610 nm and ~ 635 nm. The sharp peak at ~ 490 nm arose as a result of near band-edge emission. Deep level emission induced by several crystal defects might account for the appearance of other minor peaks. In the PL spectrum of In_2_O_3_/Fe_3_O_4_, all these peaks appear with reduced intensity. In the emission spectrum of In_2_O_3_/MoS_2_/Fe_3_O_4_, all the aforesaid peaks were even less intense. Furthermore a slight red shift was observed in the nanocomposites when compared with the emission spectra of pristine In_2_O_3_. This was consistent with the results of UV–visible absorbance spectra and could be attributed to interfacial interactions between the moieties of the nanohybrids^42–44^. The PL data therefore support the interactive integration of the moieties of the two final nanohybrids that resulted in a reduction in the recombination of charge carriers further undermined by the trapping of electrons by Fe^3+^ ions of the Fe_3_O_4_ integrant.

### Magnetic behaviour

**Figure 6:**
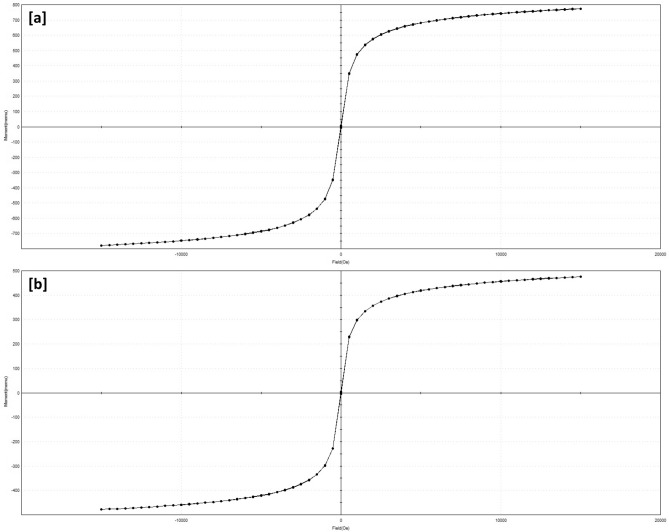
Magnetic hysteresis curves of (**a**) Fe_3_O_4_ and (**b**) In_2_O_3_/MoS_2_/Fe_3_O_4_.

In order to evaluate the response of pristine Fe_3_O_4_ and In_2_O_3_/MoS_2_/Fe_3_O_4_ towards externally applied magnetic fields for proper evaluation of their magnetic properties, the initial magnetization versus field measurements of the samples were performed by magnetometer operating at ambient temperatures, with the field sweeping from − 15,000 to 15,000 Oe following which the magnetization hysteresis curves were obtained (Fig. 6a,b). Both the samples so designed demonstrated considerable superparamagnetic nature. The magnetic saturation and coercivity (Hci) of pristine Fe_3_O_4_ (Fig. 6a) were 56.58 emu g^−1^ and 13.892 G respectively, while, In_2_O_3_/MoS_2_/Fe_3_O_4_ exhibited magnetic saturation of 31.98 emu g^−1^ and coercivity (Hci) of 10.768 G (Fig. 6b).

### Evaluation of the impacts of operating parameters on the photocatalytic decomposition of esomeprazole

#### Influence of photocatalyst loading

**Figure 7:**
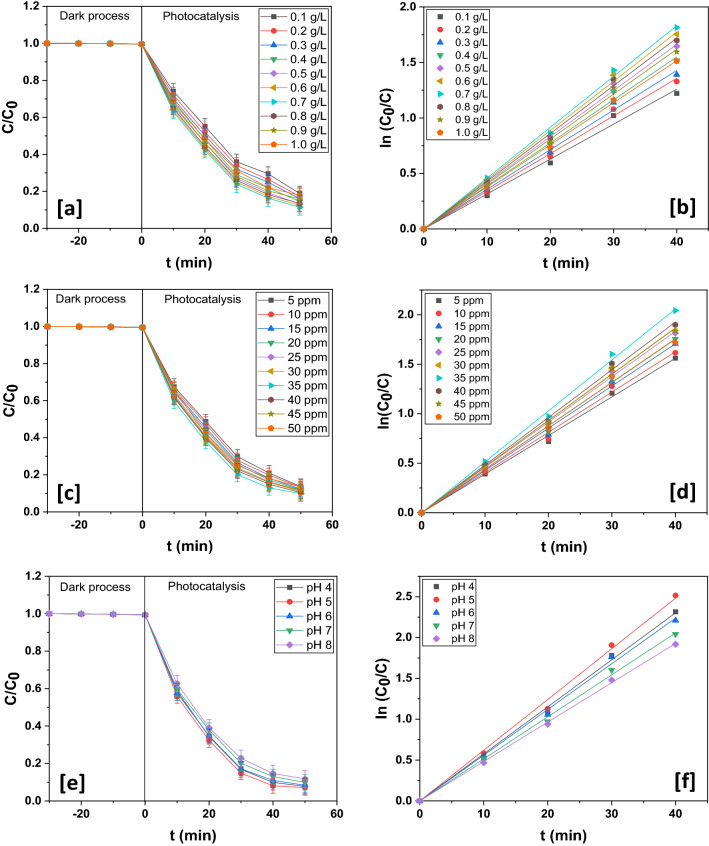
(**a**) Photodegradation dynamic curves of esomeprazole at different catalyst loading In_2_O_3_/MoS_2_/Fe_3_O_4_ and (**b**) its corresponding kinetics. (**c**) Photodegradation dynamic curves of esomeprazole at different esomeprazole concentration loading over /MoS_2_/Fe_3_O_4_ and (**d**) its corresponding kinetics. (**e**) Photodegradation dynamic curves of esomeprazole at different pH over In_2_O_3_/MoS_2_/Fe_3_O_4_ and (**f**) its corresponding kinetics.

The influence of the catalyst content required for the photodecomposition of esomeprazole was put to scrutiny. For this, other operating parameters, such as initial esomeprazole concentration and initial pH were held constant respectively at 25 ppm and 7 and the catalyst content was altered across the range 0.1–1 gL^−1^. The optimum quantity of 0.7 gL^−1^ of In_2_O_3_/MoS_2_/Fe_3_O_4_ (Fig. 7a) was required for accomplishing esomeprazole photodecomposition of ~ 88.61 ± 2.14% at a velocity constant of ~ 0.04578 min^−1^ (Fig. 7b and Table 2). A gradual rise in esomeprazole photodecomposition with increase in catalyst dosage was recorded until the optimal limit beyond which photodecomposition declined. With increase in catalyst loading, solution opacity rises. With consequent increased turbidity and light scattering, there occurred increased shortening of photon path-length. As a result, photons could not traverse deep into the suspension. This prevented activation of the entire catalyst surface that eventually led to subdued generation of active radicals, thereby reducing the photodecomposition efficiency. Optimization of photocatalyst content is absolutely necessary to avert inordinate use of it in the experiment.

**Table 2:** Photodegradation chart of esomeprazole over In_2_O_3_/MoS_2_/Fe_3_O_4_ at different catalyst doses.

Dose of catalyst (gL^−1^)	% degradation	k (min^−1^)	R^2^
0.1	81.16 ± 1.98	0.03145	0.99629
0.2	82.61 ± 2.05	0.03392	0.99803
0.3	83.96 ± 2.03	0.03569	0.99780
0.4	85.44 ± 1.99	0.03875	0.99759
0.5	86.78 ± 1.87	0.4155	0.99818
0.6	87.57 ± 2.12	0.04433	0.99881
0.7	88.61 ± 2.14	0.04578	0.99875
0.8	86.56 ± 2.11	0.04298	0.99876
0.9	84.03 ± 2.16	0.04046	0.99863
1.0	82.19 ± 2.13	0.03789	0.99956

#### Influence of initial esomeprazole concentration

The influence of initial concentration of esomeprazole was investigated next. For this the optimal catalyst content was taken and the pH was held constant at 7. The photodecomposition was carried out using initial concentration of the pharmaceutical varying from 5 to 50 ppm. Over 0.7 gL^−1^ of In_2_O_3_/MoS_2_/Fe_3_O_4_ photocatalyst, esomeprazole decomposition first increased with drug concentration, became highest at 35 ppm and then declined (Fig. 7c). At this optimal drug concentration, In_2_O_3_/MoS_2_/Fe_3_O_4_ achieved a photodecomposition efficiency of ~ 90.12 ± 2.06%with a velocity constant of ~ 0.05144 min^−1^ following pseudo-first order kinetics (Fig. 7d and Table 3). There could be three reasons for a fall in photodecomposition efficiency beyond the optimal value of pharmaceutical concentration. One, with increase in pharmaceutical concentration, the ability of photons to penetrate deep into the solution decreases leading to lower efficiency of photodecomposition. Two, for photodecomposition of the drug beyond the optimal value, extra amount of photocatalyst would be required which would again enhance solution opacity. Three, as the pharmaceutical concentration is increased, greater number of active sites on the photocatalyst surface was occupied by the pharmaceutical species eventually evicting surface-adsorbed hydroxide ions and oxygen molecules from the active sites. The generation of active radicals by the photocatalyst thus suffered a setback leading to reduced photodecomposition efficiency.

**Table 3:** Photodegradation chart of esomeprazole over In_2_O_3_/MoS_2_/Fe_3_O_4_ at different concentrations of esomeprazole.

Concentration of esomeprazole (ppm)	% degradation	k (min^−1^)	R^2^
5	86.33 ± 2.07	0.03899	0.99856
10	86.73 ± 1.97	0.04064	0.99790
15	87.72 ± 2.05	0.04266	0.99858
20	88.12 ± 2.16	0.04393	0.99874
25	88.61 ± 2.14	0.04578	0.99875
30	89.06 ± 2.17	0.04658	0.99872
35	90.12 ± 2.06	0.05144	0.99890
40	89.58 ± 2.26	0.04813	0.99882
45	88.28 ± 2.19	0.04668	0.99889
50	86.56 ± 2.09	0.04381	0.99863

#### Influence of initial pH

The influence of initial pH was examined using fixed initial drug concentration of 35 ppm and an optimum catalyst dosage (0.7 gL^−1^). There was initially an intensification of photodecomposition behaviour as pH was increased but after a pH of 5, there was a slight reduction in photodecomposition efficiency (Fig. 7e). At the optimal pH, an esomeprazole photodecomposition efficiency of ~ 92.92 ± 2.01% was observed at a pseudo-first order velocity constant of ~ 0.06208 min^−1^ (Fig. 7f and Table 4). Esomeprazole stays predominantly in protonated form under acidic conditions, however, as the pH rises, deprotonated, anionic form of esomeprazole predominates^45,46^. As initial pH rises, there hydroxide ions increasingly accumulates over the photocatalyst surface leading to an intensified electrostatic effect that would render the photocatalyst surface repulsive to the deprotonated esomeprazole, which would exert a negative effect on the photodecomposition.

**Table 4:** Photodegradation chart of esomeprazole over In_2_O_3_/MoS_2_/Fe_3_O_4_ at different pH values.

pH	% degradation	k (min^−1^)	R^2^
4	92.24 ± 2.05	0.05757	0.99849
5	92.92 ± 2.01	0.06208	0.99811
6	91.62 ± 2.05	0.05596	0.99840
7	90.12 ± 2.06	0.05144	0.99890
8	88.26 ± 2.20	0.04822	0.99640

#### Influence of contact time

**Figure 8:**
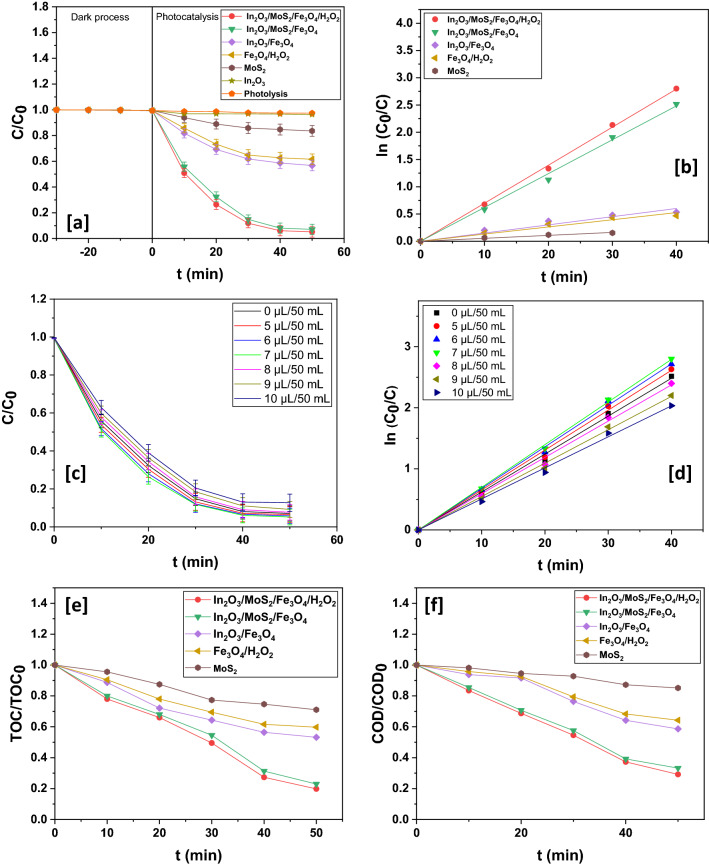
(**a**) Photodegradation dynamic curves of esomeprazole and (**b**) kinetics for different catalysts. (**c**) Photodegradation dynamic curves of esomeprazole at different H_2_O_2_ doses over In_2_O_3_/MoS_2_/Fe_3_O_4_ and (**d**) its corresponding kinetics. (**e**) Plot of TOC/TOC_0_ vs. time. (**f**) Plot of COD/COD_0_ vs. time.

To determine the influence of contact time, optimum values of operating parameters were employed (Fig. 8a,b). Photodecomposition experiments were executed using 0.7 gL^−1^ of In_2_O_3_/MoS_2_/Fe_3_O_4_ alongside a fixed initial pharmaceutical concentration of 35 ppm and an initial pH of 5. After 50 min no noticeable photocatalytic activity could be observed. This near stoppage in the photocatalytic decomposition of esomeprazole could be due to saturation of the surface of the nano-scaled hybrid photocatalysts so that no active sites are available for further photocatalytic decomposition.

#### Influence of H_2_O_2_

H_2_O_2_ addition has been known to augment decomposition of organic species^47–50^. Further, the presence of Fe_3_O_4_ as a moiety in the nanohybrid of In_2_O_3_/MoS_2_/Fe_3_O_4_ could also enable activation of a Fenton-like phenomenon upon incorporation of H_2_O_2_ in the photodecomposition reaction mixture. Therefore, it was absolutely necessary to explore the influence of H_2_O_2_ on the photocatalytic activities of the fabricated nano-scaled integrated photocatalysts. This was done by varying the dosage of H_2_O_2_ in the range of 5–10 µL/50 mL of the reaction mixture. Over the integrated nano-photocatalyst system, decomposition activity initially registered a spike till a value of 7 µL/50 mL H_2_O_2_ dosage and then it displayed a minor slump with further addition of H_2_O_2_ (Fig. 8c,d). Upon illumination with visible light, there occurred self-disintegration of H_2_O_2_ producing ^·^OH radicals that accounted for the initial surge in decomposition behaviour.

3 $${\text{H}}_{2} {\text{O}}_{2} + {\text{h}}\upvartheta \to \, 2^{\cdot} {\text{OH}}$$

Additionally, Fe on the surface of the nano-photocatalysts could trigger augmented generation of ^·^OH radicals via Fe^III^/Fe^II^ redox couple.

4 $${\text{Fe}}^{{{\text{III}}}} + {\text{ H}}_{2} {\text{O}}_{2} \to {\text{Fe}}^{{{\text{III}}}} - {\text{H}}_{2} {\text{O}}_{2}$$

5 $${\text{Fe}}^{{{\text{III}}}} - {\text{H}}_{2} {\text{O}}_{2} \to {\text{Fe}}^{{{\text{II}}}} + {\text{HO}}_{2}^{ \cdot } + {\text{H}}^{ + }$$

6 $${\text{Fe}}^{{{\text{III}}}} + {\text{HO}}_{2}^{ \cdot } \to {\text{Fe}}^{{{\text{II}}}} + {\text{O}}_{2} + {\text{H}}^{ + }$$

7 $${\text{Fe}}^{{{\text{II}}}} + {\text{H}}_{2} {\text{O}}_{2} \to {\text{Fe}}^{{{\text{III}}}} +^{ \cdot } {\text{OH}} + {\text{OH}}^{ - }$$

Ligand displacement leads to the formation of a complex designated by Fe^III^-H_2_O_2_. This complex thereafter undergoes intermolecular electron transfer generating HO_2_^·^ radicals with the simultaneous reduction of Fe^III^ to Fe^II^. HO_2_^·^ radicals thus generated undergo reaction with Fe^III^ causing further reduction of Fe^III^ to Fe^II^. Eventually, Fe^II^ and H_2_O_2_ react together to produce ^·^OH radicals that makes unselective attacks on the drug species causing its disintegration.

Under alkaline conditions, there could also be the formation of a less reactive ferryl ion species (Fe^IV^-O)^51^.

8 $${\text{Fe}}^{{{\text{II}}}} + {\text{H}}_{2} {\text{O}}_{2} \to {\text{Fe}}^{{{\text{IV}}}} - {\text{O}} + {\text{H}}_{2} {\text{O}}$$

Self-absorption of ^·^OH radical by H_2_O_2_ could account for the minor decline in the decomposition efficiency at H_2_O_2_ doses beyond the optimal value.

9 $${\text{H}}_{2} {\text{O}}_{2} +^{ \cdot } {\text{OH}} \to {\text{HO}}_{2}^{ \cdot } + {\text{H}}_{2} {\text{O}}$$

Decomposition profile over In_2_O_3_/MoS_2_/Fe_3_O_4_ alongside H_2_O_2_ is demonstrated in Table 5. At an optimal H_2_O_2_ dosage of 7 µL/50 mL, a photodecomposition efficiency of ~ 94.17 ± 2.03% was registered over In_2_O_3_/MoS_2_/Fe_3_O_4_ at pseudo-first order velocity constant of ~ 0.06798 min^−1^.

**Table 5:** Photodegradation chart of esomeprazole over In_2_O_3_/MoS_2_/Fe_3_O_4_ at different doses of H_2_O_2_.

Dose of H_2_O_2_ (µL/50 mL)	% degradation	k (min^−1^)	R^2^
0	92.92 ± 2.01	0.06208	0.99811
5	93.55 ± 2.23	0.06534	0.99814
6	94.17 ± 2.03	0.06798	0.99881
7	94.78 ± 2.08	0.06980	0.99948
8	92.30 ± 2.05	0.05935	0.99756
9	90.77 ± 2.20	0.05465	0.99852
10	87.28 ± 2.25	0.05079	0.99817

#### Performances of different photocatalysts

Figure 8a and Table 6 display photocatalytic performances of different photocatalysts while Fig. 8b represents the corresponding photodegradation kinetics. Photolysis carried out in absence of catalysts showed no appreciable decomposition of the pharmaceutical. The pristine samples also showed inferior decomposition capacities. Pristine In_2_O_3_ could barely affect appreciable photodecomposition of esomeprazole. Pristine MoS_2_ brought about ~ 16.43 ± 2.01% of esomeprazole decomposition at pseudo-first order velocity constant of ~ 0.00054 min^−1^. The binary nanohybrid of In_2_O_3_/Fe_3_O_4_ attained ~ 43.26 ± 1.51% esomeprazole decomposition obeying pseudo-first order kinetics with a velocity constant of ~ 0.01503 min^−1^. Esomeprazole photodecomposition over In_2_O_3_/MoS_2_/Fe_3_O_4_ was considerably remarkable as the nano-scale integrated photocatalyst was able to bring on ~ 92.92 ± 2.01% of the drug decomposition within 50 min at a pseudo-first order velocity constant of ~ 0.06208 min^−1^ with TOC reduction and COD reduction up to ~ 77.06% and ~ 66.71% respectively (Fig. 8e,f). In_2_O_3_/MoS_2_/Fe_3_O_4_ functioned ~ 2.15 times better than the binary nanohybrid on the efficiency front. As discussed earlier, the presence of H_2_O_2_ had an augmenting effect on photocatalysis as decomposition efficiency In_2_O_3_/MoS_2_/Fe_3_O_4_ went up to ~ 94.17 ± 2.03%.

**Table 6:** Photodegradation chart of esomeprazole over various catalysts.

Samples	% degradation	k (min^−1^)	% TOC removal	% COD reduction	R^2^
In_2_O_3_/MoS_2_/Fe_3_O_4_ + H_2_O_2_	94.78 ± 2.08	0.06980	80.17	70.82	0.99948
In_2_O_3_/MoS_2_/Fe_3_O_4_	92.92 ± 2.01	0.06208	77.06	66.71	0.99811
In_2_O_3_/Fe_3_O_4_	43.26 ± 1.51	0.01503	46.77	41.33	0.97721
Fe_3_O_4_ + H_2_O_2_	38.44 ± 1.52	0.01315	40.23	35.74	0.98151
MoS_2_	16.43 ± 2.01	0.00054	28.96	14.86	0.99161

#### Reusability of the fabricated ternary nanohybrids

Reusability is absolutely vital for the proper appraisal of the stability, longevity and efficacy of a fabricated nano-scaled photocatalyst. Therefore, after the complete process of esomeprazole photodecomposition, the hybrid nano-scaled photocatalyst was subjected to reusability tests.

**Figure 9:**
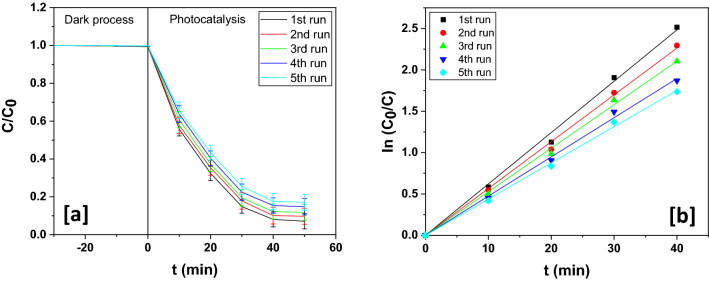
(**a**) Photodegradation dynamic curves of esomeprazole for five consecutive runs over In_2_O_3_/MoS_2_/Fe_3_O_4_ and (**b**) its corresponding kinetics.

**Figure 10:**
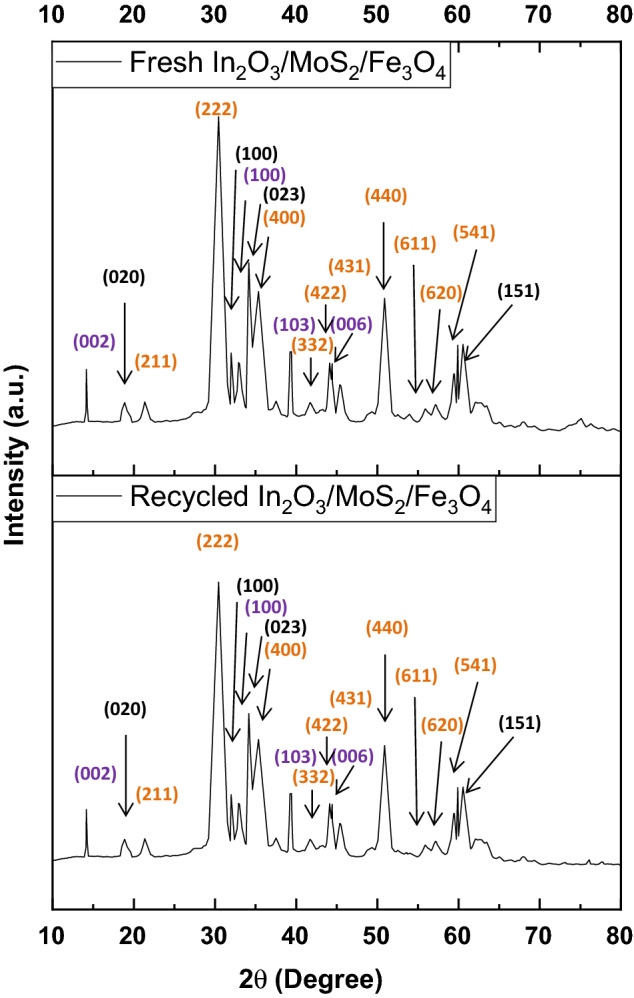
XRD pattern for fresh and recycled In_2_O_3_/MoS_2_/Fe_3_O_4_.

The photocatalyst was first magnetically recovered. It was further subjected to regeneration by washing with deionized water and ethanol and then drying in an oven at 70 °C. The photocatalyst was used again for the decomposition of esomeprazole and for five consecutive times and it demonstrated fairly consistent decomposition behaviour (Fig. 9a,b) without any noteworthy slump in photocatalytic performance (Table 7). The slight fall of photodecomposition efficiency registered by the photocatalyst in this course could be attributed to the dislodgement of the moieties from the integrated photocatalyst system. XRD analysis of the photocatalyst was done before and after (Fig. 10) the fifth cycle of the pharmaceutical decomposition for determination of variations in crystallinity. No appreciable variations in crystallinity of In_2_O_3_/MoS_2_/Fe_3_O_4_ were noted before and after photocatalytic performance. The crystallographic planes that appeared in the unused photocatalyst also made their presence in the XRD plot of the recycled photocatalysts suggesting retention of crystallographic structure of the photocatalyst after five consecutive runs of photodecomposition experiment. This implied that the integrated photocatalyst was stable, durable and efficient.

**Table 7:** Photodegradation chart of esomeprazole over In_2_O_3_/MoS_2_/Fe_3_O_4_ for five cycles.

Runs	% degradation	k (min^−1^)	R^2^
1st	92.92 ± 2.01	0.06208	0.99811
2nd	90.43 ± 2.13	0.05658	0.99863
3rd	88.23 ± 2.12	0.05262	0.99869
4th	85.24 ± 2.18	0.04739	0.99853
5th	82.88 ± 2.11	0.04378	0.99889

#### Scavenger experiments

**Figure 11:**
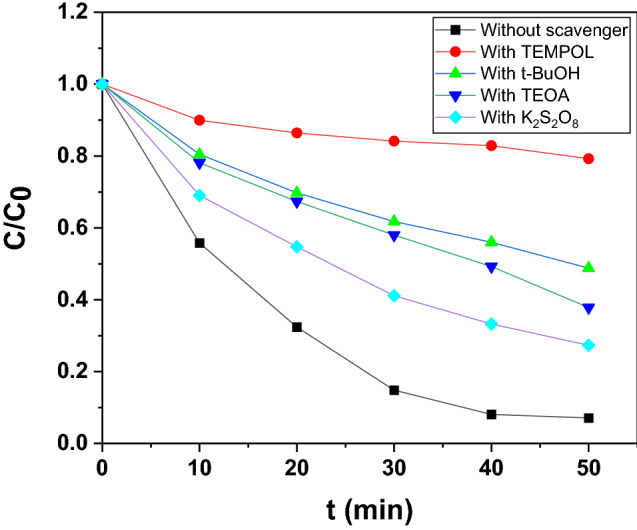
Effect of scavengers on photodegradation.

The influences of incorporating radical scavengers were also determined. The objective behind this study was to properly asses the roles played by the reactive species such as ·OH, O_2_^−·^, e^−^, and h^+^. Scavengers were added during photodecomposition experiments for trapping of reactive species for ascertaining the species that actively trigger/s disintegration of esomeprazole in aqueous medium. 4-hydroxy-2,2, 6,6- tetramethylpiperidinyloxy (TEMPOL) is a O_2_^−·^ scavenger and is widely known to quench the radical leading to a slowdown of the photocatalytic kinetics driven by O_2_^−·^ radicals. The addition of TEMPOL had a marked negative effect on the photocatalytic decomposition dynamics of esomeprazole decomposition over In_2_O_3_/MoS_2_/Fe_3_O_4_. With insertion of 1 mmol of TEMPOL, the esomeprazole photodecomposition efficiency dropped to ~ 20.77%. Likewise, tert-butanol (t-BuOH) is a ^·^OH scavenger and has been reported to inhibit photocatalysis instigated by ^·^OH radicals. Indeed, photodecomposition was appreciably but moderately inhibited upon incorporation of 1 mmol of t-BuOH. The insertion of t-BuOH brought on a drastic slowdown in the photodecomposition dynamics of esomeprazole decomposition over In_2_O_3_/MoS_2_/Fe_3_O_4_. Efficiency dropped to ~ 51.21% over In_2_O_3_/MoS_2_/Fe_3_O_4_. The hole scavenger, triethanolamine TEOA and the electron scavenger, K_2_S_2_O_8_ didn’t have any significant disruptive effects on the photodecomposition dynamics of esomeprazole decomposition evidently suggesting the minor roles played by holes and electrons. Therefore, it emerged from the study that ROS such as O_2_^−·^ and ^·^OH were chiefly involved in triggering photodecomposition of aqueous esomeprazole over the photocatalyst. Figure 11 shows the photodecomposition behaviour in presence of the aforementioned scavengers over In_2_O_3_/MoS_2_/Fe_3_O_4_.

#### Mechanism of photodecomposition over ternary nanohybrid photocatalysts

**Figure 12:**
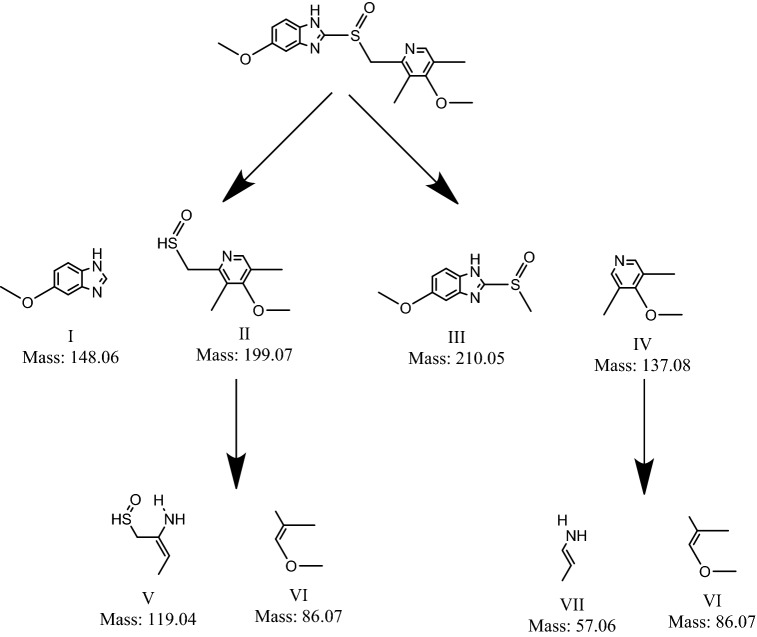
Schematic diagram of the plausible mechanism of degradation.

In view of the aforementioned experimental results, the mechanism of photocatalysis by In_2_O_3_/MoS_2_/Fe_3_O_4_ is illustrated in Fig. 12. The CB of In_2_O_3_ (~ − 0.6 eV versus SHE) being more negative than that of MoS_2_ (~ − 0.5 eV versus SHE), there was a migration of photogenerated electrons from the CB of In_2_O_3_ to that of MoS_2_ following which there occurred interaction of these electrons with adsorbed O_2_ species to form O_2_^−·^^46,47^. With a band gap of 1.91 eV, the VB of MoS_2_ (~ + 1.39 eV versus SHE) would be placed higher than that of In_2_O_3_ (∼ + 2.2 eV versus SHE)^52,53^. This would drive holes from the VB of In_2_O_3_ to that of MoS_2_. The interaction of holes with H_2_O would enable production of ^·^OH. The involvement of Fe^III^/Fe^II^ redox couple would cause additional generation of ^·^OH radicals. Such interactions to produce reactive species have been previously reported over Fe_3_O_4_ containing photocatalytic systems^18,19,54–56^. In our experiment, we admit, it is difficult to prove the formation of such an intermediate hypervalent Fe-IV species during photodegradation mechanism. However, certain works have cited the possible generation of such species^57–59^. These reactive species caused decomposition of esomeprazole as illustrated below:

10 $${\text{In}}_{2} {\text{O}}_{3} + {\text{visible}}\;{\text{light}} \to {\text{In}}_{2} {\text{O}}_{3} \left( {{\text{e}}^{ - }_{{{\text{CB}}}} } \right) + {\text{In}}_{2} {\text{O}}_{3} \left( {{\text{h}}^{ + }_{{{\text{VB}}}} } \right)$$

11 $${\text{In}}_{2} {\text{O}}_{3} \left( {{\text{e}}^{ - }_{{{\text{CB}}}} } \right) + {\text{MoS}}_{2} \to {\text{In}}_{2} {\text{O}}_{3} + {\text{MoS}}_{2} \left( {{\text{e}}^{ - }_{{{\text{CB}}}} } \right)$$

12 $${\text{O}}_{2} + {\text{In}}_{2} {\text{O}}_{3} \left( {{\text{e}}^{ - }_{{{\text{CB}}}} } \right) \to {\text{O}}_{2}^{ - \cdot } + {\text{ In}}_{2} {\text{O}}_{3}$$

13 $${\text{h}}^{ + }_{{{\text{VB}}}} + {\text{H}}_{2} {\text{O}} \to {\text{H}}_{2} {\text{O}}_{2} + 2{\text{H}}^{ + }$$

14 $${\text{H}}_{2} {\text{O}}_{2} + {\text{e}}^{ - } \to {\text{OH}}^{ - } +^{ \cdot } {\text{OH}}$$

15 $${\text{Fe}}^{{{\text{III}}}} + {\text{e}}^{ - } \to {\text{Fe}}^{{{\text{II}}}}$$

16 $${\text{Fe}}^{{{\text{II}}}} + {\text{O}}_{2} \to {\text{Fe}}^{{{\text{III}}}} + {\text{O}}_{2}^{ - \cdot }$$

17 $${\text{Fe}}^{{{\text{III}}}} + {\text{h}}^{ + } \to {\text{Fe}}^{{{\text{IV}}}}$$

18 $${\text{Fe}}^{{{\text{IV}}}} + {\text{OH}}^{ - } \to {\text{Fe}}^{{{\text{III}}}} +^{ \cdot } {\text{OH}}$$

19 $${\text{O}}_{2}^{ - \cdot } /^{ \cdot } {\text{OH}} + {\text{Esomeprazole}} \to {\text{Degraded}}\;{\text{metabolites}}$$

**Figure 13:**
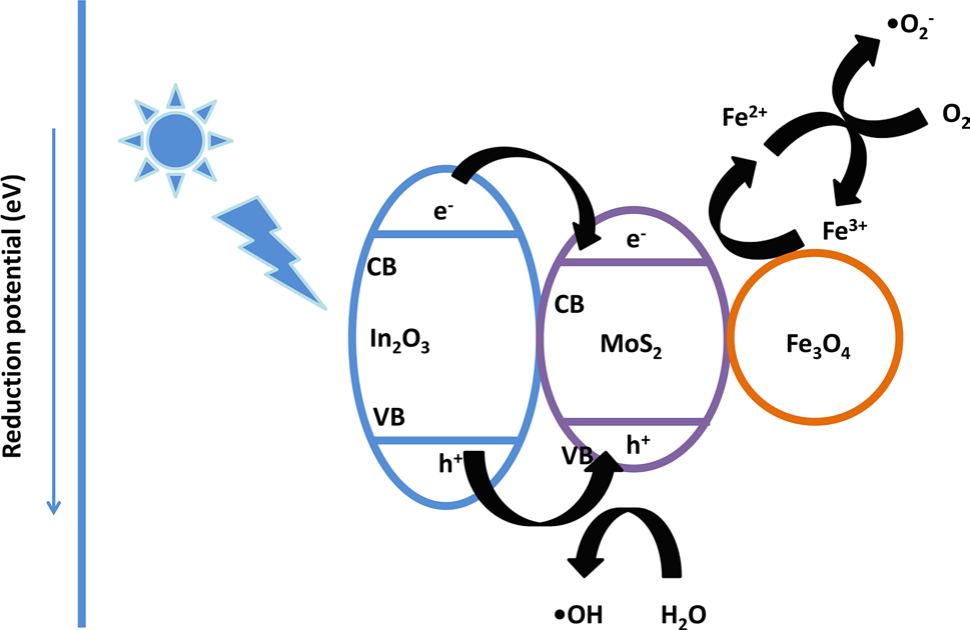
Plausible disintegration pathway of esomeprazole.

Additionally, there might also occur transference of electrons from the conduction band of Fe_3_O_4_ to that of MoS_2_ eventually leading to the generation of the aforementioned reactive species. Furthermore, HRLCMS conducted at a halfway stage of light assisted disintegration of esomeprazole over In_2_O_3_/MoS_2_/Fe_3_O_4_, made it possible to outline a plausible pathway of esomeprazole decomposition (Fig. 13). Figure S4 shows liquid chromatograms obtained for esomeprazole decomposition over the two ternary nanohybrid photocatalyst and Figures S5 display the mass spectra corresponding to various intermediates.

#### Influence of inorganic salts

The influence exerted by inorganic salts on the photocatalytic decomposition of organic contaminants has been investigated in several studies^60–62^. The impacts of inorganic ions and cations on the photodecomposition efficiency of esomeprazole decomposition over the two ternary photocatalysts were studied.

**Figure 14:**
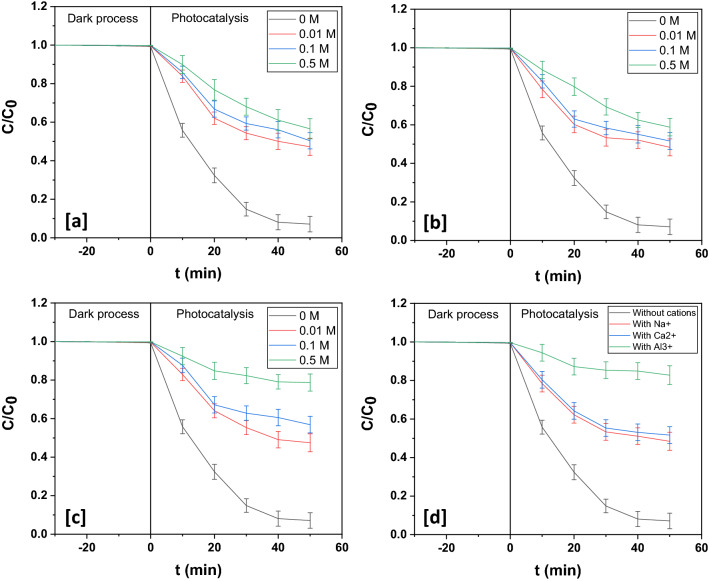
Photodegradation dynamic curves of esomeprazole in presence of different concentrations of (**a**) chloride, (**b**) sulfate and (**c**) bicarbonate over In_2_O_3_/MoS_2_/Fe_3_O_4_. (**d**) Photodegradation dynamic curves of esomeprazole in presence of different cations over In_2_O_3_/MoS_2_/Fe_3_O_4_.

In this study it emerged that chloride ions hindered the progress of photocatalytic decomposition of aqueous esomeprazole over In_2_O_3_/MoS_2_/Fe_3_O_4_. Decomposition dynamics after incorporation of chloride ions in the photocatalysis reaction vessel is illustrated in Fig. 14a. The data reproduced in Table S1 indicates the gradual suppression of esomeprazole decomposition with increase in chloride concentration. This is ascribed to the quenching of hydroxyl radicals by chloride ions. Further, due to preferential adsorption of chloride ions at active sites of the photocatalysts, there might occur encircling of the photocatalysts by chloride ions leading to further slowdown of photocatalysis. Figures S6a depicts the kinetics of these photodecomposition experiments. The equations underneath describe the happenings in presence of chloride ions:

20 $${\text{Cl}}^{ - } + {\text{h}}^{ + } \to^{ \cdot } {\text{Cl}}$$

21 $${\text{Cl}}^{ - } +^{ \cdot } {\text{Cl}} \to {\text{Cl}}_{2}^{ - \cdot }$$

22 $${\text{Cl}}^{ - } +^{ \cdot } {\text{OH}} \to^{ \cdot } {\text{Cl}} + {\text{OH}}^{ - }$$

The chloride ions first undergo adsorption on photocatalyst surface and thereafter following reaction with photogenerated holes are oxidized to chlorine free radicals. These radicals further interact with electrons to get reduced to chloride ions. The photodecomposition inhibiting power of chloride ions could be assigned to its non-oxidizable nature. Greater concentration of chloride ions leads to severely intense adsorption and interactions with photo-holes as a result of which esomeprazole decomposition declines.

Next the influence of sulfate ions on the pharmaceutical degradation was examined. It is obvious from Table S2 that the introduction of sulfate ions also had a negative effect on the photocatalytic decomposition of esomeprazole over the ternary photocatalyst. With rise in concentration of sulfate ions, photodegradation gets impeded. This is also illustrated in Fig. 14b. In comparison with chloride ions, the bulkier sulfate ions could be more intensely adsorbed on the photocatalyst surface and its bivalent nature would only reinforce the process. After adsorption, the sulfate ions would undergo interactions with reactive species as illustrated below:

23 $${\text{SO}}_{4}^{2 - } + {\text{h}}^{ + } \to {\text{SO}}_{4}^{ - \cdot }$$

24 $${\text{SO}}_{4}^{2 - } +^{ \cdot } {\text{OH}} \to {\text{SO}}_{4}^{ - \cdot } + {\text{OH}}^{ - }$$

25 $${\text{SO}}_{4}^{ - \cdot } + {\text{e}}^{ - } \to {\text{SO}}_{4}^{2 - }$$

The above reactions readily explain the intense retarding effect exerted by sulfate ion on esomeprazole degradation. Figure S6b depicts the kinetics of these photodecomposition experiments.

The influence of bicarbonate on photocatalysis ions was then studied. A decline in photodegradation of esomeprazole over both the ternary photocatalysts was yet again observed. The two-fold mechanism of competing for reactive species and blockage of active sites on photocatalyst surface by adsorption of bicarbonate ions might account for the impeding effect exerted by the presence of the anion. The following second order reaction explains the interaction:

26 $${\text{HCO}}_{3}^{2 - } +^{ \cdot } {\text{OH }} \to {\text{H}}_{2} {\text{O}} + {\text{CO}}_{3}^{ - \cdot }$$

There is a gradual decrease in the decomposition efficiency with increase in the concentration of bicarbonate ions as illustrated in Table S3. Figures (14c and S6c) portray degradation profiles over the ternary photocatalyst.

Several metal cations are usually present in wastewater and therefore their impacts on the decomposition of organic contaminant were studied. Na^+^, Ca^2+^ and Al^3+^ would not obstruct photocatalytic degradation because they are in their stable oxidation state. In this study to examine the effects of these cations, esomeprazole degradation was carried out in presence of 0.01 M of Na_2_SO_4_, CaSO_4_ and Al_2_(SO_4_)_3_. The data thus generated is presented in Table S4 while degradation dynamics are portrayed in Figs. (14d and S6d). Quite evidently there is a hindrance offered by these salts in varying degrees to photocatalytic degradation although this obstruction could be due to the suppressing influence by sulfate ions of the salts. Highest retarding effect was exerted by aluminum salt and this could be due to adsorption of Al^3+^ ions at the active sites of the photocatalysts.

#### Influence of organic acids, other organic compounds and environmental waters

**Figure 15:**
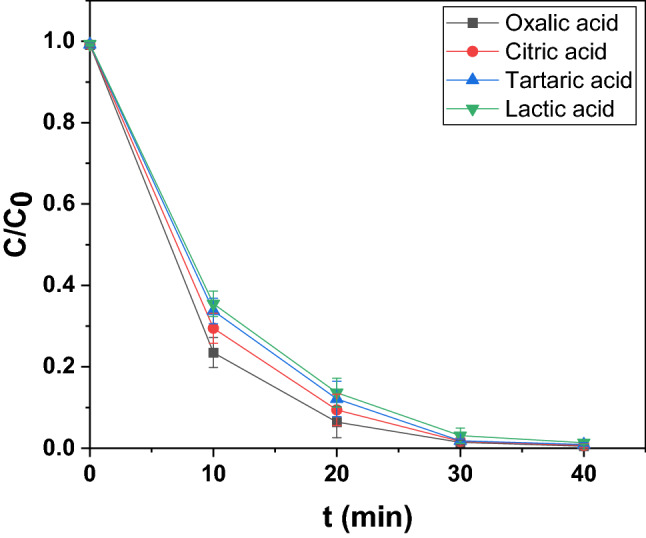
Photodegradation dynamic curves of esomeprazole in presence of different organic acids over In_2_O_3_/MoS_2_/Fe_3_O_4_.

The decomposition of living organism generates organic acids that are found to accumulate in aquatic environment and therefore the influences of a few organic acids like oxalic acid, citric acid, tartaric acid and lactic acid on photodecomposition of esomeprazole was investigated. 0.5 mmolL^−1^ of each of these acids was incorporated in the photocatalysis reaction vessel and their effects on photocatalytic activity was recorded. All of them accelerated photodecomposition and also the photodecomposition yield. In presence of oxalic acid with two -COOH groups in which one -OH of a -COOH influenced by ortho-COOH functions like α-OH group the highest photodecomposition yield and the maximum magnitude of velocity constant were registered. Citric acid having three -COOH groups and an α-OH group came next followed by tartaric acid (two -COOH groups and two α-OH groups) and then lactic acid (one -COOH group and one α-OH group). While investigating the effect of pH on photodegradation, although a slight initial increase up till pH 5 followed by a slight decrease in the yield was noted, there wasn’t any reduction in the optimal contact time of the reaction. However, in presence of organic acids, there was a marked acceleration of photodecomposition that led to a considerable reduction of optimal contact time to 40 min. Hence, the effect of pH in this phenomenon may be ignored. The number of -COOH and α-OH groups, however, could be correlated to this acceleration of photodecomposition^63,64^. Although precise elaboration of parameters affecting this phenomenon will require further work along this direction. The decomposition profile is shown in Fig. 15 and its corresponding kinetics in Figure S7. Table S5 demonstrates the photocatalytic performances in presence of the organic acids.

The influence of organic moieties such as, acetone, humic acid sodium salt (HAS) and sodium dodecyl sulfate (SDS) on esomeprazole photodegradation over In_2_O_3_/MoS_2_/Fe_3_O_4_ was also investigated. With remarkable solvent properties acetone is frequently used in industrial applications and everyday life. Humic acids and SDS are also frequently found in wastewater.

As a photosensitizer, acetone has the repute to enhance photodegradation besides also exerting a decelerating effect. Acetone-photosensitization may proceed through the route delineated below:

27 $${\text{CH}}_{3} {\text{COCH}}_{3} + {\text{h}}\upvartheta \to {\text{CH}}_{3} {\text{CO}}^{ \cdot } +^{ \cdot } {\text{CH}}_{3}$$

28 $${\text{CH}}_{3} {\text{COCH}}_{3} + {\text{h}}\upvartheta \to {\text{CH}}_{3} {\text{COH}}_{2} {\text{C}}^{ \cdot } + {\text{H}}^{ \cdot }$$

29 $${\text{O}}_{2} + {\text{CH}}_{3} {\text{COH}}_{2} {\text{C}}^{ \cdot } \to {\text{CH}}_{3} {\text{COH}}_{2} {\text{COO}}^{ \cdot }$$

30 $${\text{CH}}_{3} {\text{COH}}_{2} {\text{COO}}^{ \cdot } + {\text{CH}}_{3} {\text{COCH}}_{3} \to {\text{CH}}_{3} {\text{COH}}_{2} {\text{COOH}} +^{ \cdot } {\text{CH}}_{2} {\text{COCH}}_{3}$$

31 $${\text{CH}}_{3} {\text{COH}}_{2} {\text{COOH}} \to {\text{CH}}_{3} {\text{COH}}_{2} {\text{CO}}^{ \cdot } +^{ \cdot } {\text{OH}}$$

**Figure 16:**
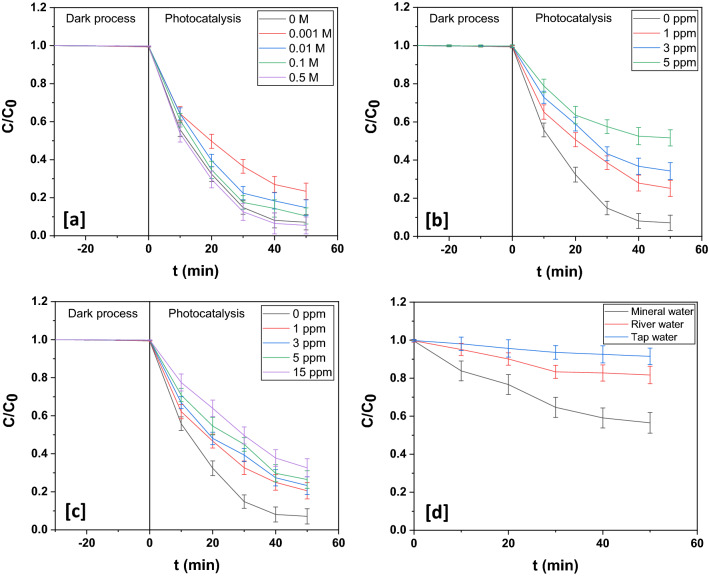
Photodegradation dynamic curves of esomeprazole photodegradation in presence of different concentrations of (**a**) acetone, (**b**) HAS and (**c**) SDS over In_2_O_3_/MoS_2_/Fe_3_O_4_. Photodegradation dynamic curves of esomeprazole photodegradation in different water matrices over In_2_O_3_/MoS_2_/Fe_3_O_4_.

In very low doses of acetone (0.001 M), the suppressing effect showed up. However, as the acetone concentration increased, photosensitization took over producing ^·^OH radicals that led to enhanced esomeprazole decomposition. The retarding drag exerted by acetone could be due to the decomposition of acetone that competed with esomeprazole decomposition. Table S6 shows the results obtained from these experiments, while Figs. (16a and S8a) portray the corresponding degradation profiles.

Table S7 shows the influence of HAS on esomeprazole decomposition over In_2_O_3_/MoS_2_/Fe_3_O_4_. In both the cases, HAS retarded the rate of decomposition. Besides, lowering the light intensity through water, HAS quenches hydroxyl radicals and also deactivates photocatalyst surface, thereby impeding the process of photocatalytic decomposition of water pollutants. Figures (16b and S8b) depict the corresponding degradation profiles. Retarding effect of SDS upon esomeprazole decomposition over the ternary photocatalyst was observed. Table S8 shows the influence of SDS on esomeprazole decomposition over In_2_O_3_/MoS_2_/Fe_3_O_4_. The probable reasons could be the formation of micelles around the drug species and interference by sulfate ions engendered by SDS photolysis. However, in this study, SDS concentrations below its critical micelle concentration (0.0085 molL^−1^ at 303.15 K) were used and so, the slump in photocatalytic activity upon incorporation of SDS could be chiefly ascribed to competitive adsorption and suppression by sulfate ions. Figures (16c and S8c) depict the corresponding degradation profiles.

Finally, the influence of three different environmental water samples on photocatalytic decomposition of esomeprazole over the ternary photocatalyst was monitored. All these water samples had negative impact on esomeprazole photocatalytic decomposition. Maximum photocatalytic activity was observed in mineral water with TOC less than 0.3 ppm. River water with TOC of 5.2 ppm showed better result than tap water with TOC of 3.4 ppm. Decomposition ability of the photocatalysts diminishes in these samples of environmental waters primarily due to attenuation of light through these media and the cumulative effect arising out of the presence of different mineral species and organic substances in them. Furthermore, lower photocatalytic activity in tap water than in river water could be due to the greater presence of minerals in the former than the latter. Table S9 shows the influence of various water samples on esomeprazole decomposition over In_2_O_3_/MoS_2_/Fe_3_O_4_. Figures (16d and S8d) depict the corresponding degradation profiles.

## Conclusion

The present study is geared towards making an assessment of the photocatalytic activities of two novel ternary nanohybrids thus obtained vis-à-vis decomposition of aqueous esomeprazole and the interactive influences exerted by operating parameters, co-existing substances and environmental water samples. The target pollutant underwent ~ 92.92 ± 2.01% decomposition over In_2_O_3_/MoS_2_/Fe_3_O_4_ at a brisk pseudo-first order velocity constant of 0.06208 min^−1^ within 50 min. The introduction of an optimum dose of 7 µL/50 mL of H_2_O_2_ augmented photodegradation. Decomposition efficiency attained over In_2_O_3_/MoS_2_/Fe_3_O_4_ went up to 94.78 ± 2.08% at a velocity constant of 0.06980 min^−1^. Augmented production of ^·^OH radicals from H_2_O_2_ and by a Fenton like phenomenon could give rise to this intensification of photocatalytic decomposition. Inorganic anions being scavengers of ^·^OH radicals had pronounced negative impact on decomposition of esomeprazole by photocatalysis over In_2_O_3_/MoS_2_/Fe_3_O_4_. Of all the cations used, Al^3+^ ions exerted the highest retarding effect because of its adsorption at the active sites of the photocatalysts. In very low doses of acetone (0.001 M), the suppressing effect due to its predominant competitive decomposition was evident. But with rise in acetone concentration increased, photosensitization took over producing ^·^OH radicals that reinforced esomeprazole decomposition. Organic acids like oxalic acid, citric acid, tartaric acid and lactic acid markedly increased photodecomposition yield and pseudo-first order velocity constant. Both HAS and SDS induced marked fall in the photocatalytic decomposition efficiency. Environmental water samples negatively affected photocatalytic performance of the ternary photocatalyst chiefly due to the combined effect exerted by inorganic and organic moieties. The ternary photocatalyst of In_2_O_3_/MoS_2_/Fe_3_O_4_ could be further used in esomeprazole photo-decomposition up to five consecutive cycles of the photocatalysis experiment sans any significant downturn in their performances.

## Supplementary Information

Supplementary Information
