# Supplementary material for: Novel magnetically retrievable In2O3/MoS2/Fe3O4 nanocomposite materials for enhanced photocatalytic performance
Source: Sci Rep. 2021 Mar 18;11:6379. doi: 10.1038/s41598-021-85532-8 (PMC7973746; doi:10.1038/s41598-021-85532-8)
Supplement: Supplementary file 1 — Supplementary Information [file 41598_2021_85532_MOESM1_ESM.pdf]

Electronic supplementary information

Novel magnetically retrievable $In_2O_3/MoS_2/Fe_3O_4$ nanocomposite materials for enhanced photocatalytic performance

Sauvik Raha, Md. Ahmaruzzaman*

Department of Chemistry, National Institute of Technology Silchar - 788010, Assam, India

*Email: mda2002@gmail.com

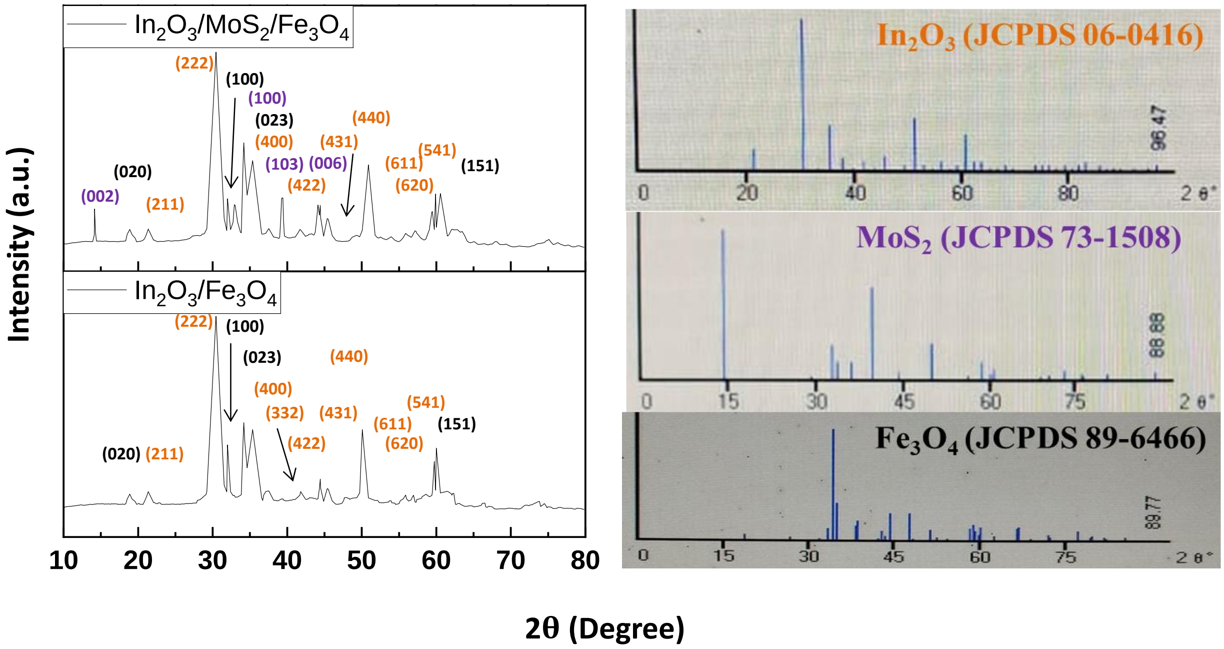

Figure S1. XRD patterns of nanocomposites with JCPDS cards.

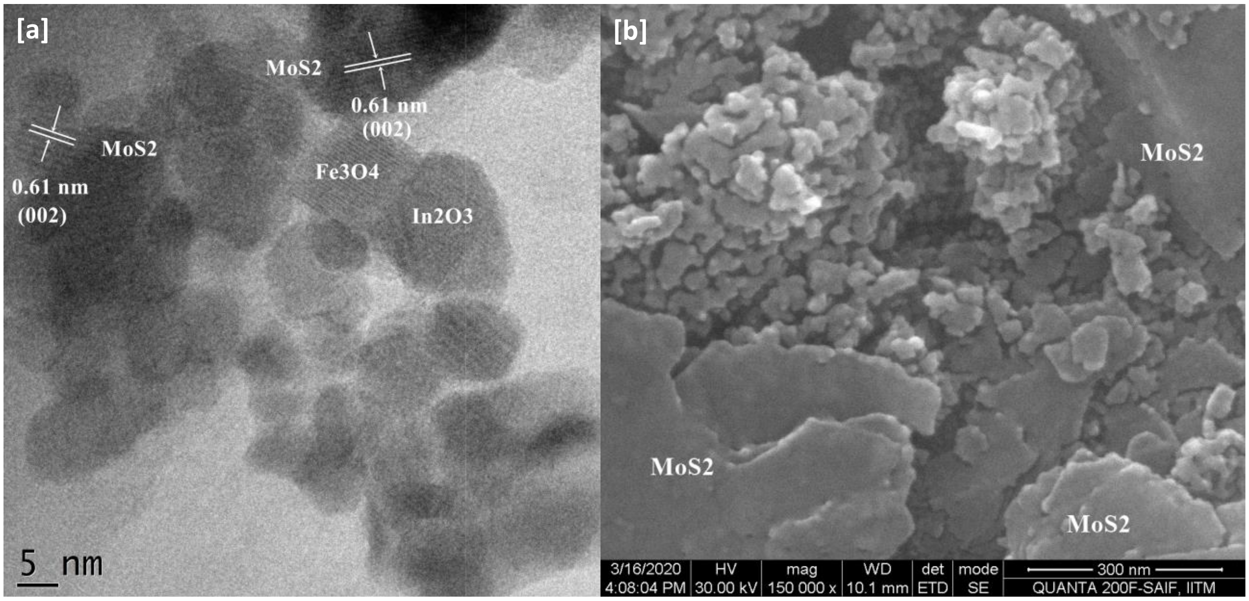

Figure S2. (a) HRTEM and (b) SEM micrographs for detection of MoS_2 nanosheets in the final nanocomposite.

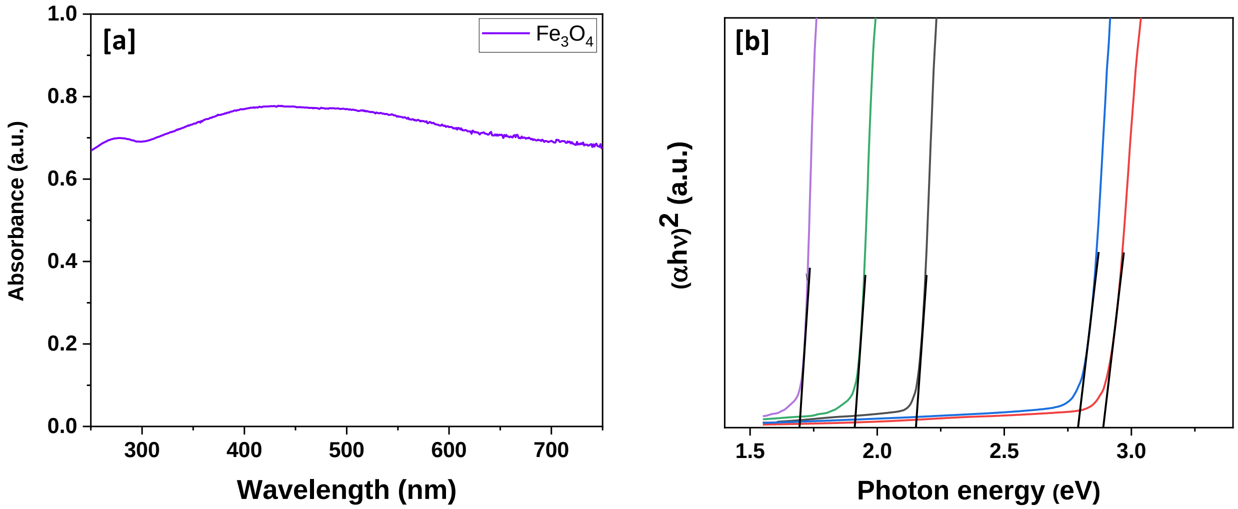

Figure S3. Enlarged diagrams of (a) absorbance curve of pristine Fe_3O_4 and (b) Tauc's plot of all samples

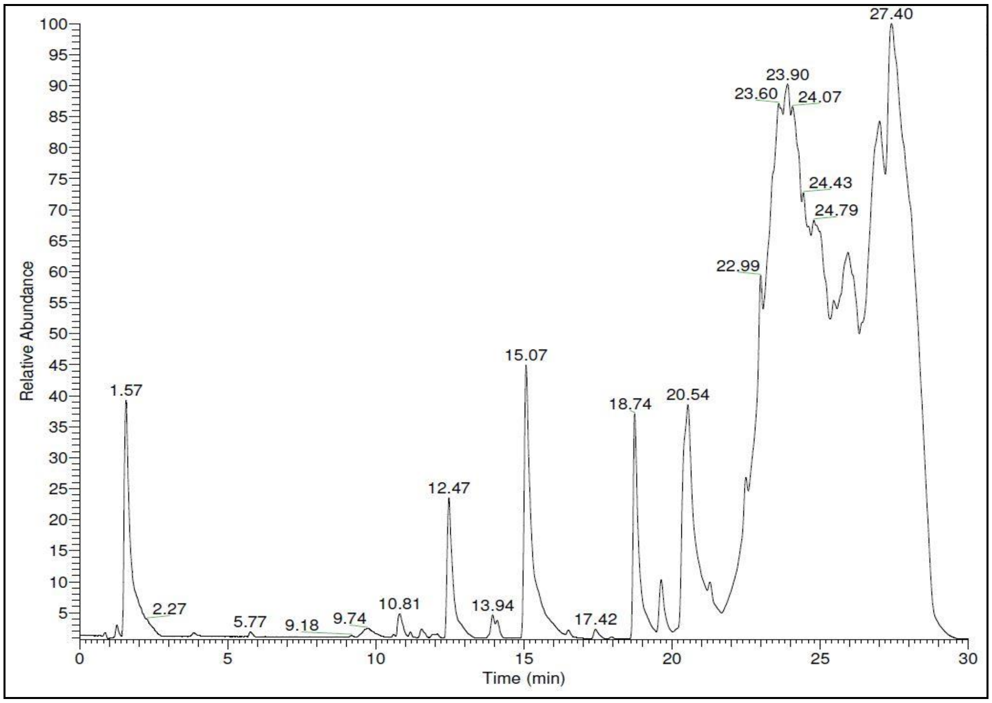

Figure S4. Liquid Chromatogram for the separation of degradation products of esomeprazole by $In_2O_3/MoS_2/Fe_3O_4$.

.

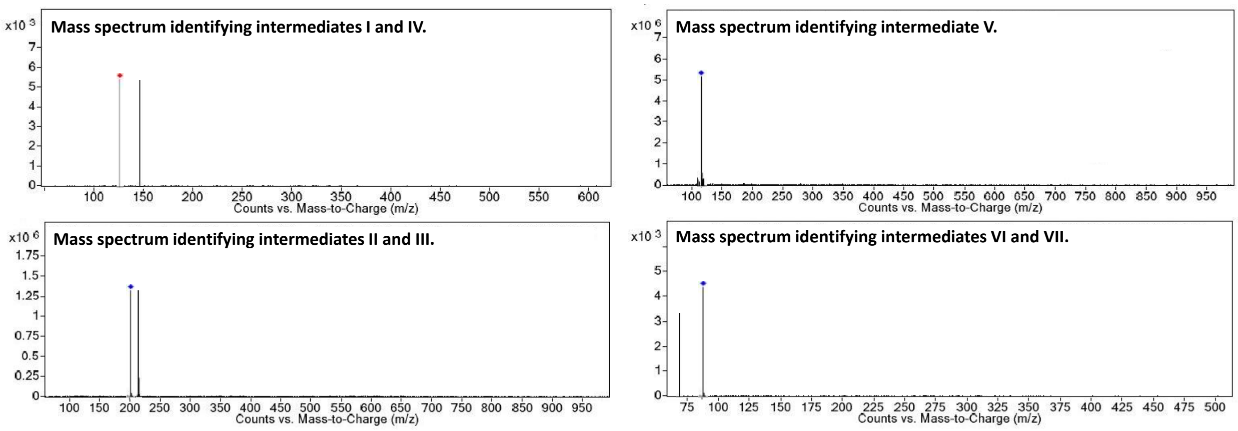

Figure S5. Mass spectra identifying various degradation products obtained during esomeprazole degradation by $In_2O_3/MoS_2/Fe_3O_4$.

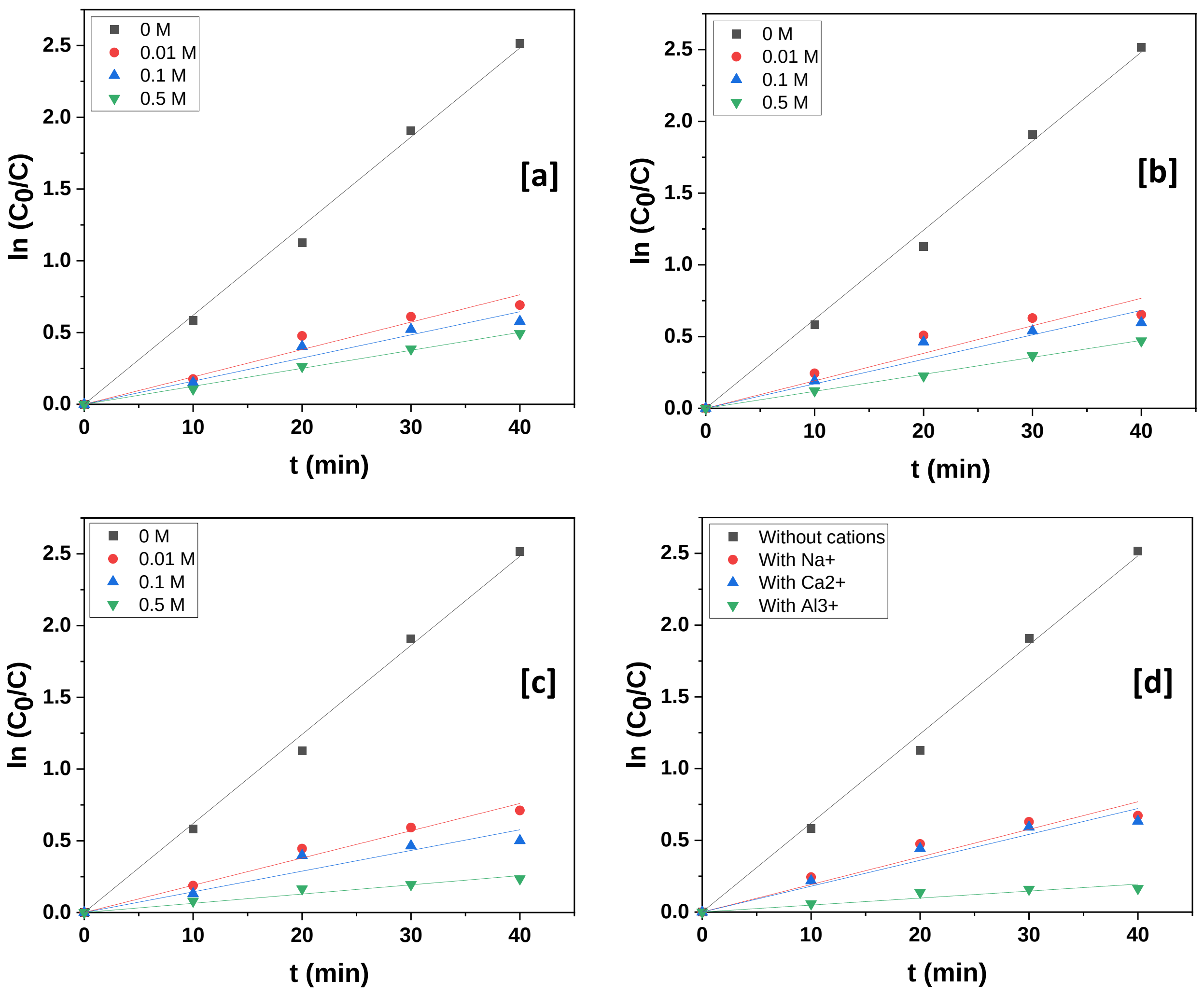

Figure S6. Kinetics of esomeprazole photodegradation in presence of different concentrations of (a) chloride, (b) sulfate and (c) bicarbonate over $In_2O_3/MoS_2/Fe_3O_4$.(d) Kinetics of esomeprazole photodegradation in presence of different cations over $In_2O_3/MoS_2/Fe_3O_4$.

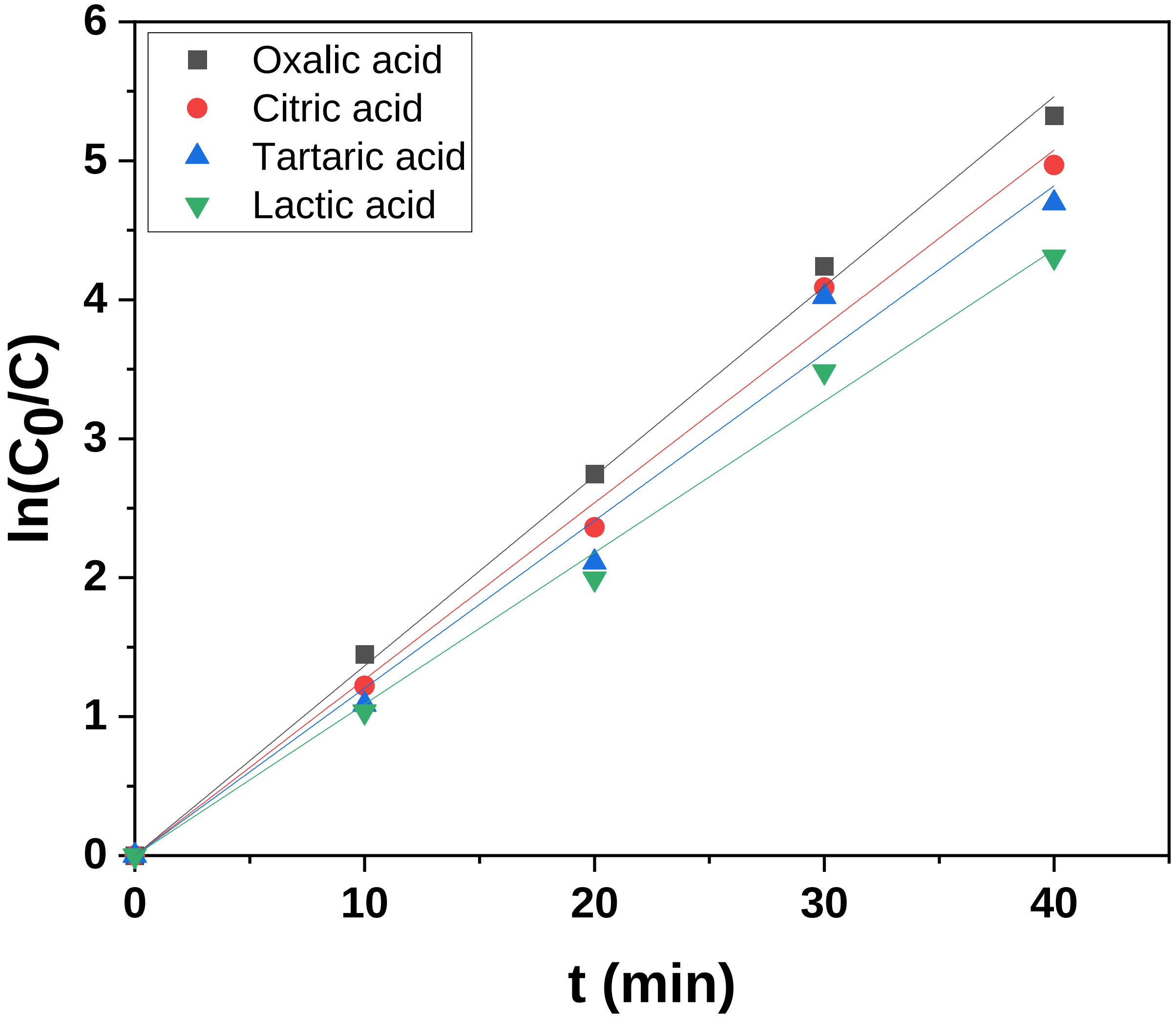

Figure S7. Kinetics of esomeprazole photodegradation in presence of different organic acids over $In_2O_3/MoS_2/Fe_3O_4$.

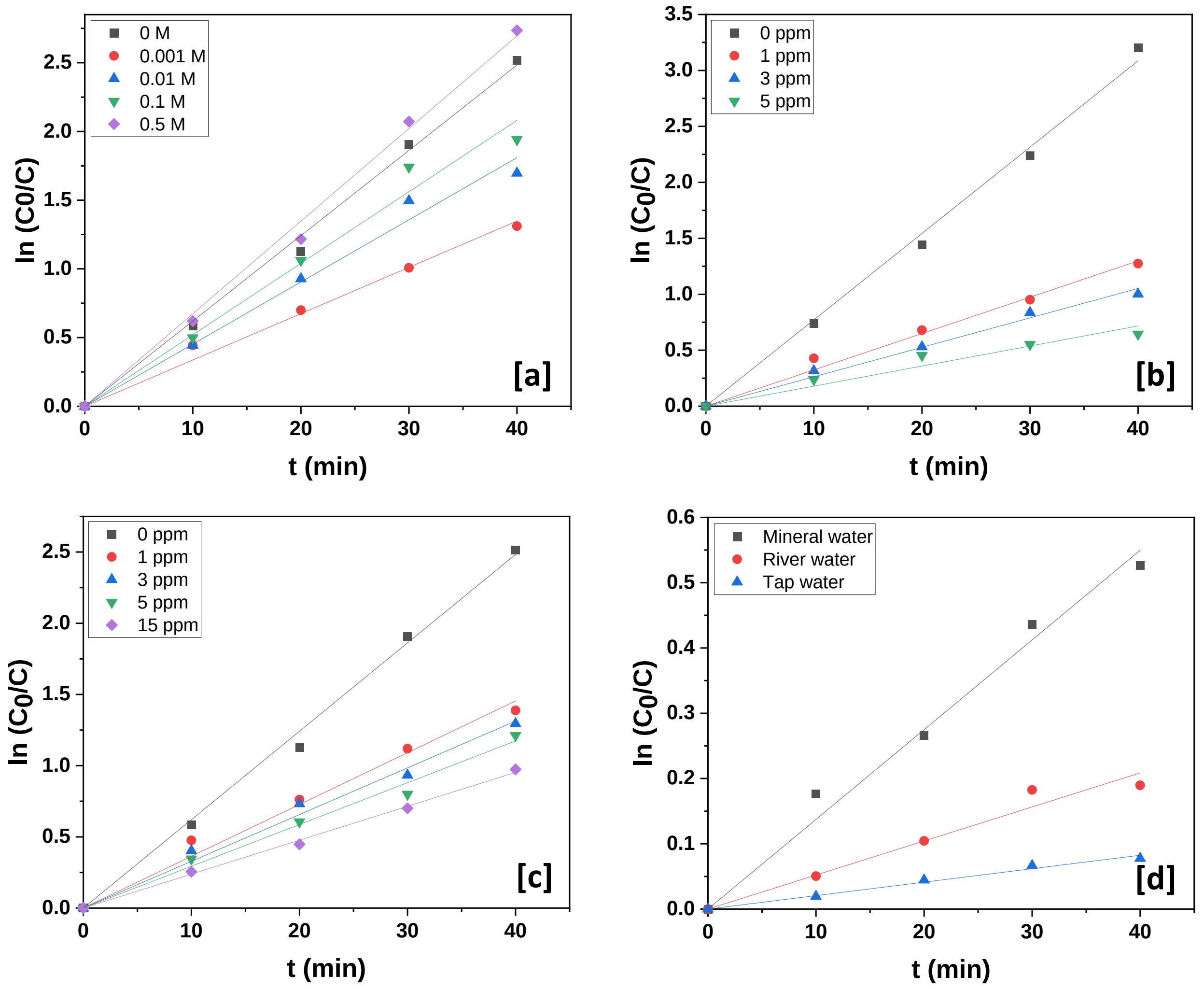

Figure S8. Kinetics of esomeprazole photodegradation in presence of different concentrations of (a) acetone, (b) HAS and (c) SDS over $In_2O_3/MoS_2/Fe_3O_4$. (d) Kinetics of esomeprazole photodegradation in different water matrices over $In_2O_3/MoS_2/Fe_3O_4$.

Table S1. Photodegradation chart of esomeprazole in presence of different concentrations of chloride over $In_2O_3/MoS_2/Fe_3O_4$

Chloride concentration (M)	% degradation	k (min^{-1})	R^2
0	92.92 ± 2.01	0.06208	0.99811
0.01	52.79 ± 2.24	0.01909	0.98213
0.1	49.63 ± 2.12	0.01613	0.98021
0.5	43.42 ± 2.63	0.01254	0.99818

Table S2. Photodegradation chart of esomeprazole in presence of different concentrations of sulphate over $In_2O_3/MoS_2/Fe_3O_4$

Sulphate concentration (M)	% degradation	k (min^{-1})	R^2
0	92.92 ± 2.01	0.06208	0.99811
0.01	51.68 ± 2.18	0.01918	0.96262
0.1	48.38 ± 2.19	0.01706	0.96758
0.5	41.21 ± 2.27	0.01185	0.99918

Table S3. Photodegradation chart of esomeprazole in presence of different concentrations of bicarbonate over $In_2O_3/MoS_2/ Fe_3O_4$

Bicarbonate concentration (M)	% degradation	k (min^{-1})	R^2
0	92.92 ± 2.01	0.06208	0.99811
0.01	52.53 ± 2.31	0.01900	0.99192
0.1	43.17 ± 2.13	0.01443	0.96338
0.5	21.29 ± 2.22	0.00644	0.97980

Table S4. Photodegradation chart of esomeprazole in presence of different cations over $In_2O_3/MoS_2/ Fe_3O_4$

Cations	% degradation	k (min^{-1})	R^2
No salt	92.92 ± 2.01	0.06208	0.99811
With Na^+	51.58 ± 2.34	0.0192	0.97467
With Ca^{2+}	48.35 ± 2.16	0.01805	0.97669
With Al^{3+}	17.24 ± 2.43	0.00488	0.95359

Table S5. Photodegradation chart of esomeprazole in presence of different organic acids over $In_2O_3/MoS_2/ Fe_3O_4$

Organic acids	% degradation	k (min^{-1})	R^2
Oxalic acid	99.52 ± 0.20	0.13656	0.99895
Citric acid	99.31 ± 0.22	0.12695	0.99684
Tartaric acid	99.09 ± 0.21	0.12051	0.99189
Lactic acid	98.66 ± 0.19	0.10902	0.99697

Table S6. Photodegradation chart of esomeprazole in presence of different concentrations of acetone over $In_2O_3/MoS_2/ Fe_3O_4$

Acetone concentration (M)	% degradation	k (min^{-1})	R^2
0	92.92 ± 2.01	0.06208	0.99811
0.001	80.33 ± 2.18	0.03370	0.99508
0.01	89.45 ± 2.28	0.04524	0.99341
0.1	95.52 ± 1.49	0.05203	0.99191
0.5	98.60 ± 0.22	0.06737	0.99776

Table S7. Photodegradation chart of esomeprazole in presence of different amounts of HAS over $In_2O_3/MoS_2/\ Fe_3O_4$

Amount of HAS (ppm)	% degradation	k (min^{-1})	R^2
0	92.92 ± 2.01	0.06208	0.99811
1	74.82 ± 2.11	0.03246	0.99495
3	65.72 ± 2.20	0.02630	0.99539
5	48.36 ± 2.12	0.01792	0.97715

Table S8. Photodegradation chart of esomeprazole in presence of different amounts of SDS over $In_2O_3/MoS_2/\ Fe_3O_4$

Amount of SDS (ppm)	% degradation	k (min^{-1})	R^2
0	92.92 ± 2.01	0.06208	0.99811
1	79.44 ± 2.14	0.03636	0.99405
3	76.73 ± 2.31	0.03282	0.99455
5	73.54 ± 2.32	0.02985	0.99471
15	67.41 ± 2.39	0.02383	0.99872

Table S9. Photodegradation chart of esomeprazole in presence of different concentrations of water matrices over $In_2O_3/MoS_2/ Fe_3O_4$

Water matrices	% degradation	k (min^{-1})	R^2
Mineral water	43.41 ± 2.71	0.01499	0.99453
River water	18.33 ± 2.29	0.00639	0.99498
Tap water	8.53 ± 2.16	0.00242	0.98748
